# Transcriptomic analysis of deceptively pollinated *Arum maculatum* (Araceae) reveals association between terpene synthase expression in floral trap chamber and species-specific pollinator attraction

**DOI:** 10.1093/g3journal/jkac175

**Published:** 2022-07-21

**Authors:** Mark A Szenteczki, Adrienne L Godschalx, Jérémy Gauthier, Marc Gibernau, Sergio Rasmann, Nadir Alvarez

**Affiliations:** Université de Neuchâtel, Institut de Biologie, 2000 Neuchâtel, Switzerland; Université de Neuchâtel, Institut de Biologie, 2000 Neuchâtel, Switzerland; Geneva Natural History Museum, 1208 Genève, Switzerland; CNRS—University of Corsica, Laboratory Sciences for the Environment (SPE—UMR 6134), Natural Resources Project, 20000 Ajaccio, France; Université de Neuchâtel, Institut de Biologie, 2000 Neuchâtel, Switzerland; Geneva Natural History Museum, 1208 Genève, Switzerland; Department of Genetics and Evolution, University of Geneva, 1211 Geneva, Switzerland

**Keywords:** Chemical ecology, GC–MS, molecular ecology, plant-pollinator interactions, RNA-seq, sesquiterpenes

## Abstract

Deceptive pollination often involves volatile organic compound emissions that mislead insects into performing nonrewarding pollination. Among deceptively pollinated plants, *Arum maculatum* is particularly well-known for its potent dung-like volatile organic compound emissions and specialized floral chamber, which traps pollinators—mainly *Psychoda phalaenoides* and *Psychoda grisescens—*overnight. However, little is known about the genes underlying the production of many *Arum maculatum* volatile organic compounds, and their influence on variation in pollinator attraction rates. Therefore, we performed de novo transcriptome sequencing of *Arum maculatum* appendix and male floret tissue collected during anthesis and postanthesis, from 10 natural populations across Europe. These RNA-seq data were paired with gas chromatography–mass spectrometry analyses of floral scent composition and pollinator data collected from the same inflorescences. Differential expression analyses revealed candidate transcripts in appendix tissue linked to malodourous volatile organic compounds including indole, *p*-cresol, and 2-heptanone. In addition, we found that terpene synthase expression in male floret tissue during anthesis significantly covaried with sex- and species-specific attraction of *Psychoda phalaenoides* and *Psychoda grisescens*. Taken together, our results provide the first insights into molecular mechanisms underlying pollinator attraction patterns in *Arum maculatum* and highlight floral chamber sesquiterpene (e.g. bicyclogermacrene) synthases as interesting candidate genes for further study.

## Introduction

Angiosperms have evolved to produce a wide array of specialized metabolites to mediate their interactions with other organisms and their environment, including volatile organic compounds (VOCs) emitted from flowers, leaves, fruits, and roots. While VOCs represent a relatively small proportion of all plant metabolomic diversity (Knudsen and Gershenzon 2020), they play important functional roles in defence against predators, pathogens, and abiotic stresses (Pichersky and Gershenzon 2002; Holopainen 2004; Irwin *et al.* 2004) and as cues for pollinator attraction (Pellmyr and Thien 1986; Knudsen and Tollsten 1993; Whitehead and Peakall 2009; Junker and Parachnowitsch 2015). Pollination, particularly by insects, appears to be a major driver of angiosperm diversification (Van der Niet and Johnson 2012; Schiestl and Johnson 2013; Hernández-Hernández and Wiens 2020). Recent research has hypothesized and partially demonstrated that floral trait diversity is the result of complex interactions between plant genomic diversity among populations, pollinator network composition, and environmental conditions—all of which vary through space and time (Thompson *et al.* 2013, 2017; Friberg *et al.* 2019). Large molecular and ecological datasets, with wide spatial and temporal coverage, are therefore still needed in order to further our understanding of the evolution of key traits such as floral scent.

Pollinator-mediated selection is known to influence floral morphology (Bröderbauer *et al.* 2013; Gervasi and Schiestl 2017) and colour (Schemske and Bradshaw 1999; Newman *et al.* 2012; Trunschke *et al.* 2021), and recent studies have also identified strong evidence for variation in VOC emissions driven by pollinator preferences. Notably, comparative community ecology data and gas chromatography–mass spectrometry (GC–MS) have been combined to identify convergent VOC bouquets in unrelated plant species with similar pollinators (Fenster *et al.* 2004; Schiestl and Johnson 2013; Junker and Parachnowitsch 2015), and divergent VOC bouquets in related species with different pollinators (Dobson *et al.* 1997; Urru *et al.* 2010; Byers *et al.* 2014; Friberg *et al.* 2019). Moreover, the discovery of biochemical pathways underlying floral VOCs has greatly accelerated by next-generation sequencing technologies such as mRNA sequencing (Dhandapani *et al.* 2017; Wong, Pichersky, *et al.* 2017; Xiao *et al.* 2019). Here, we aimed to combine these techniques, in order to understand how pollinator-mediated selection influences transcriptomic variation underlying floral scent divergence among populations of deceptively pollinated *Arum maculatum* L. (Araceae).

*A. maculatum* is a common European woodland flower with a long history of study, due to its deceptive pollination strategy which involves olfactory deception through brood-site mimicry (Schmucker 1925; Knoll 1926; Vogel 1965; Lack and Diaz 1991). Specifically, *A. maculatum* emit VOCs (e.g. 2-heptanone, indole, and *p*-cresol) during anthesis which are also present in cow manure (Kite 1995) and decomposing organic material (Gfrerer *et al.* 2021, 2022): the breeding substrates of *Psychoda phalaenoides* L. and *Psychoda* (*Psycha*) *grisescens* (Tonnoir) (Espíndola *et al.* 2011; Szenteczki *et al.* 2021). These 2 dipteran (Psychodidae) moth flies are trapped at varying frequencies across the geographic distribution of *A. maculatum* (Espíndola *et al.* 2011; Szenteczki *et al.* 2021; Gfrerer *et al.* 2021). Geographic variation in *A. maculatum* floral scent has also been observed, first in England (Kite *et al.* 1998; Diaz and Kite 2002) and France (Chartier *et al.* 2011, 2013), and recently, across most of the species distribution range (Gfrerer *et al.* 2021; Szenteczki *et al.* 2021), and may be linked to variation in pollinator attraction (Gfrerer *et al.* 2021; Szenteczki *et al.* 2021).

*A. maculatum* VOC emissions during anthesis are highly variable, and also include several sesquiterpenes and other aliphatic, aromatic, monoterpene, and nitrogen-containing minor compounds which may also contribute to pollinator attraction (Kite 1995; Gfrerer *et al.* 2021; Szenteczki *et al.* 2021). Intra- and interpopulation VOC variation mainly centers around variation in the proportion of indole to terpenes (Chartier *et al.* 2013; Szenteczki *et al.* 2021), which are respectively produced by aromatic amino acid (AAA; i.e. phenylalanine, tyrosine, and tryptophan) metabolism and terpene synthases (TPSs) via the mevalonate or methylerythritol 4-phosphate (MEP) pathways. Experiments in England with scented bait traps have shown that indole, 2-heptanone, and *p*-cresol are attractive to *P. phalaenoides* (Kite *et al.* 1998). Large-scale field studies have further demonstrated that sesquiterpenes may also play a role in differential attraction of *P. phalaenoides* and *P. grisescens* (Szenteczki *et al.* 2021) and influence fruit set size (Gfrerer *et al.* 2021).

Another recognizable feature of *A. maculatum* is its inflorescence morphology. Like other monoecious Araceae, *A. maculatum* produce densely clustered, unisexual male and female florets arranged along a central spike (the spadix), which is surrounded by a leaf-like bract (the spathe). The spathe surrounds the fertile flowers, creating a basal trap chamber with only a narrow opening at the top of the chamber, which is surrounded by a ring of hair-like sterile flowers. The scent of the floral chamber during anthesis appears to be dominated by 1 sesquiterpene, bicyclogermacrene (Kite *et al.* 1998), which was previously misidentified as germacrene B (Kite 1995). The purple colour of the appendix (the sterile apex of the spadix) and stamens is due to anthocyanin pigments (Williams *et al.* 1981). Purple pigmentation in the appendix of Araceae appears to be attractive to Sphaeroceridae pollinators (Roháček *et al.* 1990; Angioy *et al.* 2004), but it is not currently known whether it is also attractive to Psychodidae, the main pollinators of *A. maculatum*. Tradeoffs between pigmentation and VOCs could also exist; however these would be limited to benzenoids (e.g. *p*-cresol and skatole) and not terpenes, which form a proportionally larger part of the floral scent bouquet in *A. maculatum* (Chartier *et al.* 2016).

The *A. maculatum* reproductive cycle takes place over 2 days (reviewed in Gibernau 2004): on the evening of the first day, the appendix is thermogenic, heating up to 15°C above ambient air temperature. Pollinators are attracted by VOCs emitted by the appendix, and fall into the trap chamber, where they crawl over the male and female florets. No rewards are provided to pollinators within the chamber. A small amount of stigmatic secretions have been observed, but the low (9–12.5%) sucrose content suggests that this fluid is not nectar (Lack and Diaz 1991), and more likely is produced to collect pollen grains (Paiva *et al.* 2021). The following morning, after the female florets are no longer receptive, pollen is released onto the trapped pollinators. Finally, pollinators are able to escape later in the day, as the sterile hairs at the opening of the trap chamber begin to wither.

In the closely related *Arum italicum*, sesquiterpene biosynthesis appears to begin in the male florets several days prior to anthesis, but the full range of characteristic floral VOCs such as *p*-cresol and skatole are only detected on the day of anthesis, in appendix tissue (Leguet *et al.* 2014). To date, the biosynthetic pathways underlying the production of *p*-cresol and skatole have only been characterized in bacteria (Selmer and Andrei 2001; Liu *et al.* 2018), and it is unclear whether *A. maculatum* and other angiosperms use similar AAA fermentation pathways to produce these VOCs. Furthermore, tissue-specific RNA-seq of *Arum concinnatum* identified more diverse transcript expression in male floret tissue on the day of anthesis rather than in the appendix (Onda *et al.* 2015), even though *A*. *concinnatum* also emits dung-like VOCs from its appendix (Urru *et al.* 2010). Consequently, many questions remain regarding the specific biosynthetic pathways underlying *A. maculatum* VOCs, the localization of their expression, and whether mRNA expression related to VOC production varies with geographic distance or pollinator community composition.

Here, we aim to address each of the aforementioned gaps in our knowledge. Our specific objectives were to: (1) investigate variation in *A. maculatum* transcript expression between appendix and male floret tissue during anthesis, (2) use these results to identify differentially expressed transcripts putatively related to VOC biosynthesis, and (3) to characterize and compare differential expression in these transcripts across Europe, with a focus on populations with divergent pollinator attraction patterns. We predicted that during anthesis, AAA metabolism would be highly expressed in appendix tissue, while sesquiterpene synthases would be highly expressed in male floret tissue. We further expected to observe differential expression of transcripts associated with AAA metabolism and TPSs in inflorescences with dung-like (i.e. indole, 2-heptanone, and *p*-cresol) vs. sesquiterpene-dominated floral bouquets, respectively. Finally, given that the *A. maculatum* population in Forêt du Gâvre, France, appears to attract almost exclusively *P. grisescens* (Espíndola *et al.* 2011; Szenteczki *et al.* 2021), we predicted that putative locally adapted transcripts associated with particular VOCs may exist within this population. To test these predictions, we surveyed VOC variation, pollinator attraction, and mRNA expression in both appendix and male floret tissue, across most of the *A. maculatum* species distribution range.

## Materials and methods

### Sampling design

**Fig. 1.**
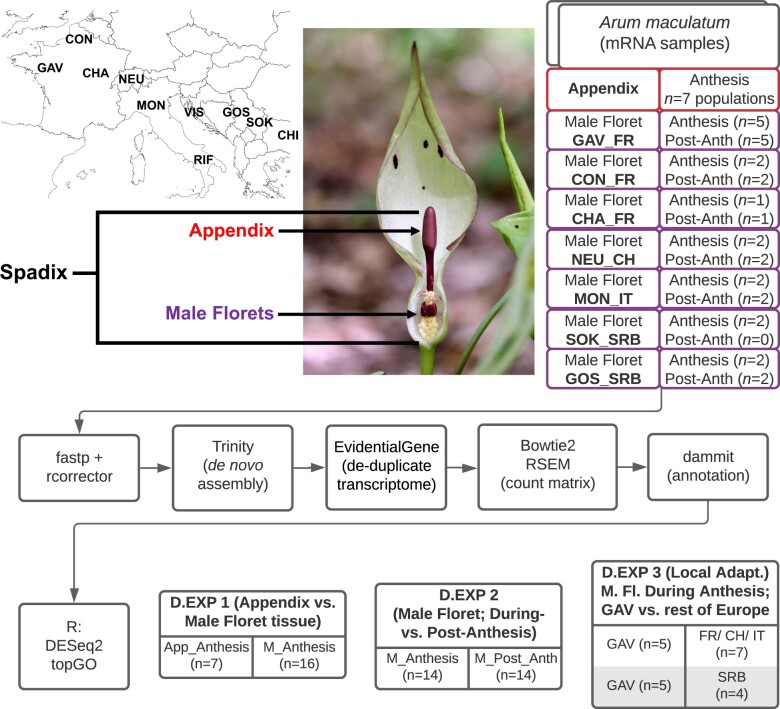
Overview of the sampling design, bioinformatic pipeline, and differential expression analyses in this study. Inset photo: *A. maculatum* inflorescence, with the lower spathe chamber dissected to reveal the male and female florets. Inset map: population codes, placed over their approximate locations.

We sampled 10 natural populations of *A. maculatum* between April and May 2019 (Fig. 1): 3 in France (Forêt du Gâvre, Conteville, and Chaumont), 1 in Switzerland (Neuchâtel), 2 in Italy (Montese and Rifreddo), 1 in Croatia (Visuć), 2 in Serbia (Gostilje and Sokobanja), and 1 in Bulgaria (Chiflik). Full details on sample data and population coordinates are given in Supplementary Table 1.

Before collecting tissue samples, we noninvasively sampled the dynamic headspace VOCs emitted by each *A. maculatum* inflorescence, following the methods detailed in (Szenteczki *et al.* 2021). Briefly, this involved placing polydimethylsiloxane coated Twister stir bars (Gerstel) in glass tubes near the appendices of *A. maculatum* inflorescences on the evening of the day of anthesis (18:00–19:00), and pumping air over them for 30 min at an air flow rate of 200 ml/min. All samples were analyzed using a gas chromatography (Agilent 7890a) mass spectrometry (Agilent 5975c) system. Desorption of the VOCs was carried out in a thermal desorption unit (TDU, Gerstel) with an initial temperature of 50°C, followed by a ramp of 60°C/min until 250°C (followed by a 3.5-min hold time). Before injection, the VOCs were cryo-focused (CIS, Gerstel) at −80°C, then released with a ramp of 12°C/min until 260°C (hold time 6 min). PTV solvent vent at 14 PSI and 50 ml/min flow was used for injection into a HP5-MS column in constant flow mode (0.9 ml/min) with helium as the carrier gas. The Mass Selective Detector (MSD) transfer line was set at 280°C. Scan mode was used for the MS acquisition with a solvent delay (1 min), over a mass range of 33–300 *m*/*z*; MS source and quadrupole temperatures were set at 230 and 150°C, respectively. Electron ionization (70 eV) was used. Major ions were recorded for each integrated peak using Agilent Chemstation, and compound identifications were derived from NIST 2.3 (library v.17) and published Kovats retention indices; all names used in our analyses should therefore be considered hypotheses. Appendix temperatures were recorded at the time of VOC sampling using FLIR thermographic imaging, and ambient air temperature was recorded using a thermistor.

Immediately following VOC sampling (i.e. during anthesis), we used sterile scalpel blades to collect 1-mm thick slices of appendix and/or male floret tissue. As a control, male floret tissue samples were also collected from inflorescences the following morning, after pollinator attraction had ended. All samples were individually preserved in RNAlater (Thermo Fischer Scientific) and stored at −80°C until extraction. After collecting each during-anthesis male floret tissue sample, we closed and sealed the small window we cut in floral chamber, so that pollinator trapping was not affected. During the morning following anthesis, we collected all insects trapped within inflorescences, preserved them in 70% ethanol and identified them to at least the suborder level. Psychodidae were further identified to species level (including sex) following taxonomic descriptions and illustrations (Ježek 1990).

### RNA-seq library preparation

All samples and tissue types were extracted using identical methods; approximately 100 mg of tissue was rinsed in sterile RNase-free water, flash frozen in liquid nitrogen, powdered in a QIAGEN TissueLyser II, and immediately extracted using a QIAGEN RNeasy Plant Mini Kit, following the manufacturer’s protocol. Extraction quality and concentration were verified using an Agilent Fragment Analyzer. Three Illumina TruSeq Stranded mRNA polyA libraries were prepared from these samples, following the manufacturer’s recommended protocol. The first library contained the appendix RNA samples, and the second and third each contained a mix of during anthesis and postanthesis male floret tissue samples from all populations. Each library was then sequenced on its own lane (i.e. 3 lanes in total) using an Illumina HiSeq 4000 (150-bp paired-end sequencing) at the Lausanne Genomic Technologies Facility (Switzerland).

### Preprocessing raw read data

We performed an initial quality filtering of all raw read files using fastp (Chen *et al.* 2018) to remove adapter sequences and polyA tails, trim reads with phred quality values below 30, and remove reads with >1 “N” bases. Then, we used rCorrector (Song and Florea 2015) to remove reads containing erroneous k-mers (25-mers). Finally, we discarded unfixable read pairs following quality filtering with *FilterUncorrectablePEfastq.py* (https://github.com/harvardinformatics/TranscriptomeAssemblyTools).

### *De novo* transcriptome assembly, annotation, and expression quantification

We merged all of the quality filtered reads and assembled a single de novo reference *A. maculatum* transcriptome (i.e. from all samples, tissue types, and populations) using Trinity v2.11.0 (Grabherr *et al.* 2011) with in silico normalization, a k-mer size of 25, minimum contig size of 200 bp, and strand-specific assembly enabled. Then, we tested 2 methods to filter out redundant transcripts with poor coding potential (i.e. minor isoforms resulting from incomplete assembly, sequencing errors, and/or heterozygosity introduced by our wide geographic sampling range): (1) CD-HIT (Li and Godzik 2006; Fu *et al.* 2012) clustering at 95 % identity with a word size of 5 and (2) the *tr2aacds* v4 pipeline from EvidentialGene (Gilbert 2019), which classifies sequences into primary, alternate, and redundant sets based on coding sequence length and similarity. When using EvidentialGene, we kept only the primary transcripts, removing the alternate and redundant isoforms. We then calculated assembly scores and assessed transcriptome completeness of both filtering methods with BUSCO v5.0.0, using the Embryophyta_odb10 dataset (Seppey *et al.* 2019).

Next, we used dammit v1.2 (Scott *et al.* 2019) to annotate our deduplicated assembly. This pipeline uses Transdecoder (https://github.com/TransDecoder/TransDecoder) to identify candidate coding regions and searches the OrthoDB, Pfam-A, Rfam, and uniref90 protein databases for transcript annotations, with an E-value threshold of 1 × 10^−5^. Unannotated transcripts were not removed from the final *A. maculatum* reference transcriptome. We also uploaded the EvidentialGene output to the Kyoto Encyclopedia of Genes and Genomes (KEGG) Automatic Annotation Server (Moriya *et al.* 2007) to generate KEGG Orthology (KO) annotations and pathway maps. Finally, we mapped the cleaned raw reads of each sample to our annotated reference transcriptome using Bowtie2 v2.3.5.1 (Langmead and Salzberg 2012) and quantified transcript expression using RSEM v1.3.2 (Li and Dewey 2011).

### Differential expression analyses

RSEM outputs were imported into R v.4.1.0 (R Core Team 2021) using *tximport* 1.20 (Soneson *et al.* 2015). Transcripts expressed below 0.75 counts per million (CPM) in at least 7 samples (i.e. the smallest sample size for a group in our analyses) were then filtered out prior to subsequent analyses in in DESeq2 1.3.2 (Love *et al.* 2014), using the *apeglm* log2fold change shrinkage estimation algorithm (Zhu *et al.* 2019). Transcripts with False Discovery Ratio (FDR)-corrected *P*-values <0.05 and log2fold changes greater than 1.0 or less than −1.0 were considered as significantly differentially expressed among groups. We further identified significantly enriched gene sets in each tissue type using Gene Ontology (GO) term analysis with the R package *TopGO* (Alexa and Rahnenfuhrer 2010) and visualized the result using the R package *rrvgo* 1.4 (Sayols 2020), an implementation of the semantic similarity-based GO summary tool REViGO (Supek *et al.* 2011).

We performed 3 sets of differential expression analyses, each addressing one of the main aims of our study. Our first comparison was between appendix (*n* = 7) and male floret (*n* = 16) tissue collected during anthesis (i.e. at the same phenological time point). Second, we compared male floret tissue collected during anthesis (*n* = 14) against their paired control samples from the morning following anthesis (*n* = 14). Paired control samples were not collected for 2 inflorescences from Sokobanja, Serbia; these individuals were excluded from this analysis. Finally, we compared transcript expression in during-anthesis male floret tissue sampled in the Forêt du Gâvre population against all other populations, to identify transcripts putatively linked to this population’s exclusive attraction of *P*. *grisescens*. To account for some of the effects of isolation by distance, we further split this analysis along the 2 main neutral genetic clusters (Espíndola and Alvarez 2011): specifically, we compared during-anthesis male floret tissue from Forêt du Gâvre (*n* = 5) against during-anthesis male floret tissue from (1) France, Switzerland, and Italy (*n* = 7) and (2) Serbia (n = 4).

### Identifying transcripts underlying VOC biosynthesis

Building on the results of our differential expression analyses, we characterized and compared the expression of metabolic pathways underlying key *A. maculatum* VOCs known to be involved in the attraction of Psychodidae pollinators (Kite *et al.* 1998): indole, *p*-cresol, 2-heptanone, and sesquiterpenes. First, we reconstructed entire metabolic pathways using automated annotations, GO terms, and KEGG pathway maps generated from (1) our complete reference transcriptome and (2) sets of significantly differentially expressed genes. Since the genes responsible for the production of 2-heptanone and *p*-cresol in *A. maculatum* are unclear and unknown respectively, we also used homology searches to identify additional candidate genes. Here, we performed BLASTp searches of genes known involved in the biosynthesis of the aforementioned VOCs in other plants and bacteria, against a database of all of our Transdecoder predicted peptide sequences using, with an *e*-value cutoff of 1 × 10^−5^. After all candidate transcripts were identified, we visualized variation in their average expression across all tissue types and populations using the R package *pheatmap* (Kolde 2019).

### Coinertia analysis—TPSs correlated with VOCs and pollinator attraction

We extracted the coding sequences and DESeq2 normalized expression of all TPSs in our transcriptome (i.e. using uniref annotations, GO/KO terms, and PFAM domains), performed a multiple sequence alignment of these sequences using Clustal Omega (Sievers *et al.* 2011), created a Maximum Likelihood phylogeny using automatic substitution model selection and default parameters in IQ-TREE (Nguyen *et al.* 2015), and visualized the result using iTOL v5 (Letunic and Bork 2021). Then, we investigated whether TPS expression was correlated with (1) total terpene emissions during anthesis or (2) the composition (i.e. sex and/or species) of trapped pollinators within inflorescences. Specifically, we performed coinertia analysis using the R packages *vegan* (Oksanen *et al.* 2020), *RVAideMemoire* (Hervé 2021), and *ade4* (Thioulouse *et al.* 2018), comparing the TPS expression matrix produced above to corresponding matrices of (1) proportional emissions of terpene VOCs and (2) proportions of Psychodidae species trapped by the same *A. maculatum* inflorescences. Prior to this analysis, the gene-level TPS expression matrix was log-transformed and scaled, while the VOC and pollinator matrices were centered log ratio transformed and scaled.

## Results

### Assembly and annotation quality

Between 27,933,352 and 66,825,898 (median: 40,354,964) paired-end reads from individual tissue samples passed all quality filtering steps (Supplementary Table 1). These were subsequently assembled into 273,346 Trinity genes, comprising 593,392 transcript isoforms. Trinity quality statistics (e.g. N50 of 1,699 bp) are given in Supplementary Table 2. EvidentialGene filtered out a larger number of transcripts from this raw assembly (Supplementary Table 3) while maintaining a higher BUSCO scores than CD-HIT clustering (e.g. 95.6% complete vs. 92.2% complete; full result in Supplementary Table 4). The EvidentialGene output was therefore annotated and used for all subsequent analyses. A total of 18,411 unique annotations were generated. A total of 17,435 annotations mapped to Eukaryota (94.7%); 12,676 of these annotations further mapped to Mesangiospermae (68.9%); a relatively small number of annotations mapped to bacterial genes (692 results; 3.8%).

A total of 49,779 transcripts remained in our expression matrix following prefiltering in DESeq2 (i.e. >0.75 CPM in at least 7 samples). Principal components analysis of filtered transcript expression (Supplementary Fig. 1) revealed highly divergent transcript expression among male floret and appendix tissue during anthesis along the first PCA axis. Along the second PCA axis, samples were further divided between 2 main geographic regions—namely, northern populations (i.e. France, Switzerland, and northern Italy) and Balkan populations (i.e. Croatia, Serbia, and Bulgaria). This regional split is consistent with the 2 main genetic clusters identified using neutral (AFLP) markers (Espíndola and Alvarez 2011).

### Differential transcript expression between appendix and male floret tissue

**Fig. 2.**
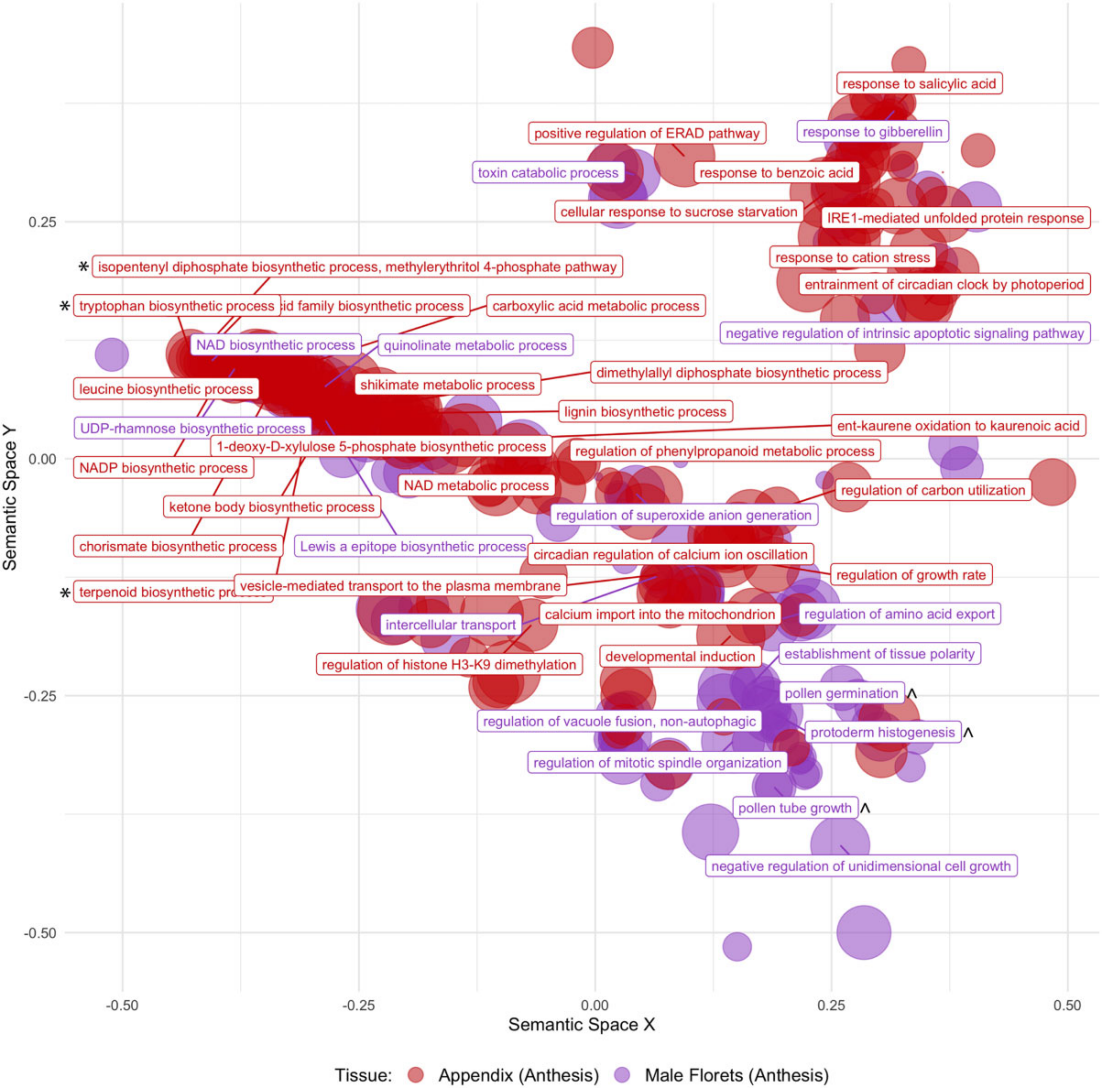
Significantly enriched GO terms when comparing *A. maculatum* appendix (*n *=* *7) and male floret (*n *=* *16) transcript expression during anthesis. Nonredundant GO terms are visualized in semantic similarity space (allowed similarity = 0.8); the full list of GO terms represented above is available in Supplementary Appendix 2. [*] indicates parent groups linked to the biosynthesis of volatile compounds and [^] indicates parent groups linked to pollen production

Differential expression analysis comparing appendix and male floret tissue (both collected during anthesis) revealed 8,683 transcripts with significantly greater expression in appendix tissue and 6,581 transcripts with significantly greater expression in male floret tissue (FDR-corrected *P*-values <0.05; log2fold change ±1), after controlling for population effects. Furthermore, we found that VOC biosynthetic activity is significantly elevated in *A. maculatum* appendix tissue during anthesis. Many major biosynthetic pathways (e.g. tryptophan/indole synthesis, terpene synthesis, and phenylpropanoid synthesis) were significantly enriched in the appendix tissue during anthesis, whereas transcripts related to pollen production were significantly enriched in male floret tissue (Fig. 2).

### Differential transcript expression in male florets during anthesis vs. postanthesis

**Fig. 3.**
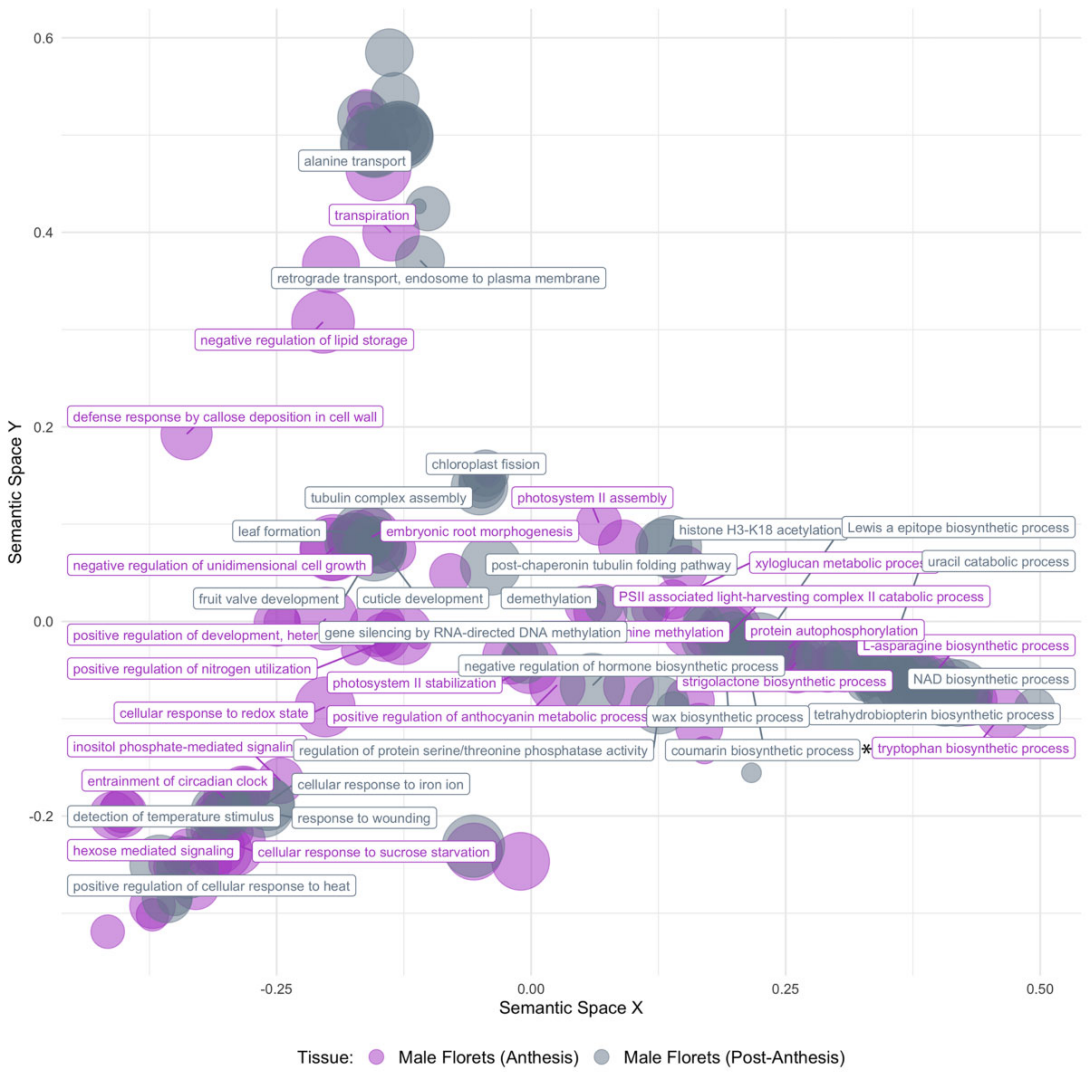
Significantly enriched GO terms when comparing *A. maculatum* transcript expression in male floret tissue during anthesis (*n *=* *14), vs. approximately 18 h after anthesis (*n *=* *14). Nonredundant GO terms are visualized in semantic similarity space (allowed similarity = 0.8); the full list of GO terms represented above is available in Supplementary Appendix 2. [*] indicates parent groups linked to the biosynthesis of volatile compounds

When comparing during-anthesis and postanthesis male floret tissue samples in a differential expression analysis that incorporated the paired nature of these samples, we identified 3,847 transcripts with significantly greater expression during anthesis and 2,920 transcripts with significantly greater expression postanthesis (FDR-corrected *P*-values <0.05; log2fold change ±1). While tryptophan (i.e. indole) biosynthesis was elevated in male florets during anthesis, we did not identify increased expression of other putative VOC biosynthetic pathways (Fig. 3). The full list of significantly enriched GO terms from both our first and second set of differential expression analyses are available in Supplementary Appendix 2.

### Differential transcript expression associated with exclusive attraction of *P. grisescens*

Our third and final set of differential expression analyses, comparing male floret tissue during anthesis among populations, revealed that (1) 84 and 9 transcripts were, respectively, differentially expressed in Forêt du Gâvre vs. all other French, Swiss, and northern Italian populations (FDR-corrected *P*-value <0.05; log2fold change ±1) and (2) 327 and 175 transcripts were, respectively, differentially expressed in Forêt du Gâvre vs. Serbian populations (FDR-corrected *P*-value <0.05; log2fold change ±1). However, no transcripts putatively linked to VOC biosynthesis were identified among these transcripts.

### Candidate genes underlying *A. maculatum* floral VOCs

Consistent with recent large-scale surveys of *A. maculatum* floral scent (Gfrerer *et al.* 2021; Szenteczki *et al.* 2021), we observed considerable within-population variation in VOC bouquet composition among our samples (Supplementary Fig. 2 and Supplementary Table 5). We further identified candidate genes putatively linked to the biosynthesis of several dung-mimicking *A. maculatum* VOCs. First, we identified transcripts in both appendix and male floret tissue homologous with tryptophan synthase alpha (TSA) subunit, which catalyzes the conversion of indole-3-glycerolphosphate to indole.

Second, we identified homologs of 2 proteins, which catalyze the degradation of tyrosine to 4-hydroxyphenyllactate: aromatic aminotransferase (ISS1) and hydroxyphenylpyruvate reductase (HPPR). Pearson correlation tests further identified several transcripts correlated with HPPR expression in appendix tissue during anthesis (Supplementary Table 6); notably, this included a putative dehydratase/shikimate dehydrogenase (Pearson *r *=* *0.988, *P < *0.001). This enzyme may further catalyze the conversion of 4-hydroxyphenyllactate to p-coumaric acid. However, we did not identify any homologs of bacterial proteins linked to the production of 4-hydroxyphenylacetate and/or *p*-cresol (Saito *et al.* 2018), such as hydroxyphenylacetate decarboxylase.

**Fig. 4.**
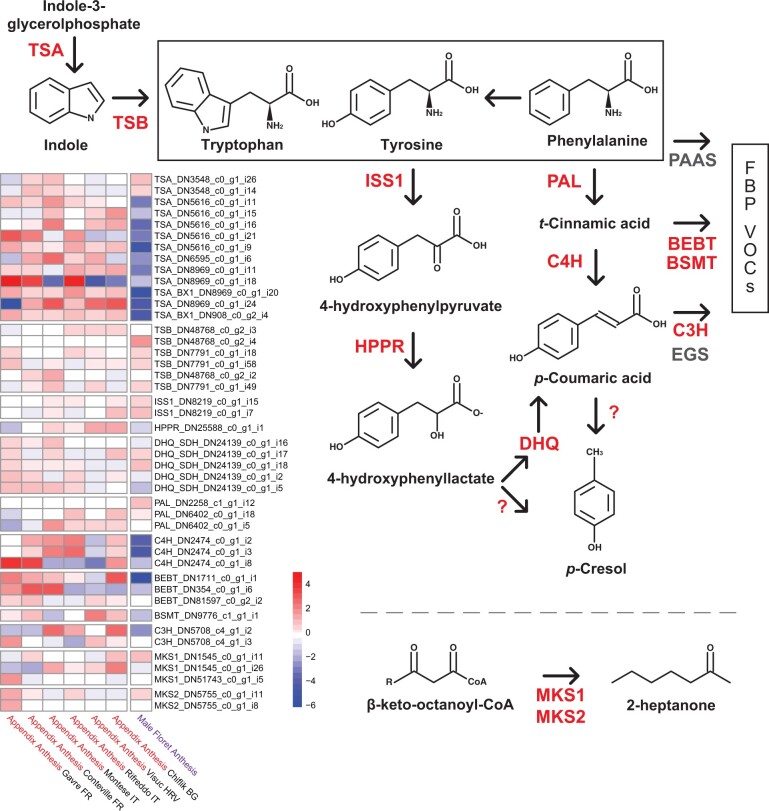
A simplified overview of key proteins involved in producing aromatic amino acid-derived *A. maculatum* VOCs, and a heatmap of their *vst*-transformed expression during anthesis. Color scale represents whether expression in a given group (i.e. tissue type and population) is above or below the transcript’s mean expression across all samples. Genes in gray are absent from the *A. maculatum* transcriptome. Gene names: BEBT = benzyl alcohol *O*-benzoyltransferase; BSMT, benzoic acid/salicylic acid carboxyl methyltransferase; DHQ, 3-dehydroquinate dehydratase/shikimate dehydrogenase; EGS, eugenol synthase; MKS, methylketone synthase; PAAS, phenylacetaldehyde synthase; TSA/B, tryptophan synthase alpha/beta subunit; ?, unknown gene(s).

Third, while phenylalanine-derived precursor molecules for several common floral benzenoid/phenylpropanoid (FBP) compounds—particularly *p*-coumaric acid—appear to be produced by *A. maculatum*, transcripts responsible for the production of benzenoid and phenylpropanoid VOCs generally appeared to be absent or weakly expressed in *A. maculatum*. Finally, we identified putative homologs of methylketone synthases (MKS1 and MKS2), which are known to be involved in 2-heptanone biosynthesis (Ben-Israel *et al.* 2009; Khuat *et al.* 2019). Heatmaps of the expression patterns for all of the aforementioned candidate transcripts are shown in Fig. 4.

**Fig. 5.**
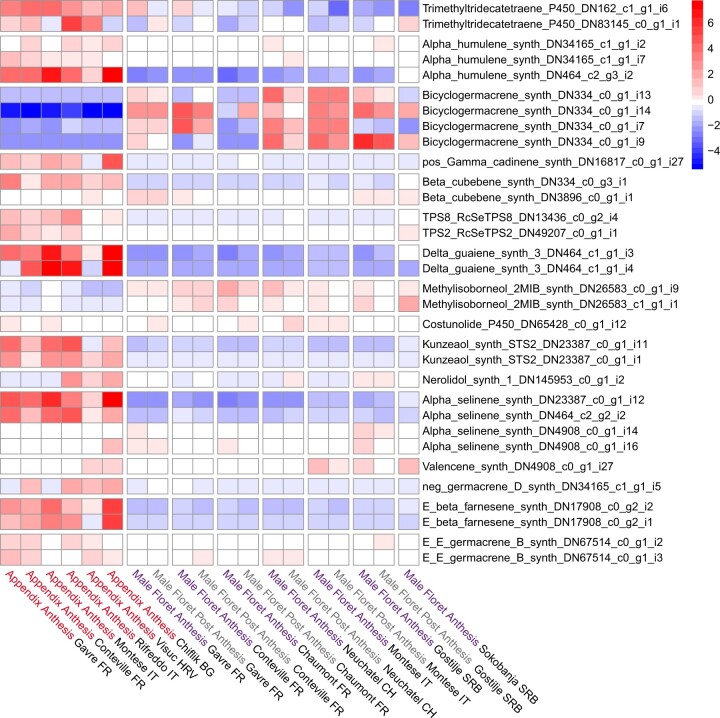
Heatmap of *vst*-transformed expression of putative terpene synthases in *A. maculatum*. Colour scale represents whether expression in a given group (i.e. tissue type, stage of anthesis, and population) is above or below the transcript’s mean expression across all samples.

We identified *A. maculatum* transcripts putatively encoding all proteins in both the mevalonate and MEP pathways. During anthesis, the MEP pathway appears to be more highly expressed than the mevalonate pathway, particularly in appendix tissue (Supplementary Fig. 3). The first enzyme of the MEP pathway, 1-deoxy-d-xylulose-5-phosphate synthase (DXS), which was highly expressed in appendix tissue, also featured the greatest isoform diversity among all terpene backbone synthesis genes. Furthermore, we identified 108 putative TPS transcripts expressed in appendix and male floret tissue during anthesis. A phylogeny of all putative *A. maculatum* TPSs is given in Supplementary Fig. 4, and expression patterns of TPS transcripts that passed our initial CPM filtering threshold are visualized in Fig. 5.

Some of the putative TPSs we identified are homologs of proteins known to produce common *A. maculatum* VOCs (e.g. humulenes and germacrenes); others, such as 2-methylisoborneol (2-MIB) synthase, catalyze the production of terpenes not previously described in *A. maculatum*. However, in the case of the latter, the transcript we identified likely encodes a novel TPS, given that we identified an uncharacterized *Colocasia esculenta* (Araceae) gene, which shares 92.5% identity with our transcript, as opposed to approximately 22.8% identity with the bacterial 2-MIB synthase (Supplementary Fig. 5). Transcripts annotated as trimethyltridecatetraene synthase (Cyt P450 92C6) may represent another such novel TPS. This gene was the only putative VOC synthase we identified that was significantly overexpressed (FDR-corrected *P*-values <0.05; log2fold change >1) in appendix tissue samples emitting high quantities of an unnamed sesquiterpene with a nonpolar Kovats Retention Index of 1681, which is known to be a strong predictor of *P*. *grisescens* attraction (Szenteczki *et al.* 2021).

### Male floret TPS transcript expression is correlated with pollinator attraction

Coinertia analyses did not identify covariation between putative TPS expression in male floret tissue, and proportional emissions of terpenes in VOC bouquets (*P = *0.964, Monte-Carlo test, 999 replicates). However, we did identify significant concordance between putative TPS expression in male floret tissue and Psychodidae pollinator composition trapped within inflorescences (*P = *0.047, Monte-Carlo test, 999 replicates); the first coinertia axis split TPS expression based on species (i.e. *P. grisescens* vs. *P. phalaenoides*), while the second coinertia axis split TPS expression based on sex (Supplementary Fig. 6). Consequently, while our coinertia analyses could not disentangle covariation in TPS expression and specific terpene VOC emissions, it appears that TPS expression in male florets is correlated with sex- and/or species-specific attraction of Psychodidae.

## Discussion

High-throughput transcriptome sequencing is rapidly advancing our understanding of the biosynthesis of floral volatile compounds. In this study, we were able to overcome some of the challenges associated with studying the genes underlying VOC biosynthesis in nonmodel systems (Wong, Pichersky, *et al.* 2017), by surveying transcript expression variation in multiple VOC-emitting floral tissues, across the Europe-wide distribution range of *A*. *maculatum* (Fig. 1). This allowed us to identify (1) elevated VOC biosynthetic activity in the appendix of *A*. *maculatum* during anthesis (Fig. 2), (2) candidate transcripts for the production of several VOCs such as *p*-cresol, 2-heptanone, and sesquiterpenes (Figs. 4 and 5), and (3) covariation between male floret TPS expression and the relative proportions of *P. phalaenoides* and *P. grisescens* trapped within inflorescences (Supplementary Fig. 6).

### Differential expression analyses

During anthesis, diverse VOC metabolic processes were significantly enriched in *A*. *maculatum* appendix tissue when compared against male floret tissue. This result is consistent with previous research, suggesting that the thermogenic appendix plays a central role in diffusing the potent VOC bouquets of Araceae (Meeuse 1975; Angioy *et al.* 2004; Barthlott *et al.* 2009). Although we expected to observe elevated TPS activity in male florets during anthesis compared to the morning following anthesis, it appears that many TPSs continued to be expressed at approximately equal levels in paired control samples collected the following morning; several factors may explain this pattern. First, given that sesquiterpenes are known to be synthesized and stored in *A. italicum* male floret tissue during the days leading up to anthesis (Leguet *et al.* 2014), mevalonate pathway and TPS expression may have peaked prior to our during-anthesis tissue sampling. However, Araceae are known to emit particularly concentrated VOC blends during anthesis (Skubatz *et al.* 1995, 1996), which should require continuous biosynthesis and emission (Widhalm *et al.* 2015). It is therefore likely that the large number of TPSs we identified in the *A. maculatum* transcriptome include those which are most relevant to pollinator attraction.

The timing of our postanthesis tissue sampling may have also influenced the above result. Postanthesis sampling occurred within 1 h after we collected and preserved all pollinators within the floral chamber; it is therefore likely that most pollinators normally would have still been trapped at this time. Both early (Dormer 1960; Prime 1960; Lack and Diaz 1991) and more recent studies (Bröderbauer *et al.* 2013) of *A. maculatum* have highlighted morphological features related to pollinator retention, namely the sterile flowers partially blocking the exit of the trap, and downward-pointing papillate cells on the inner wall of the spathe. However, continued expression of some TPS transcripts during the morning following anthesis may also highlight sesquiterpenes putatively involved in pollinator retention within the trap chamber. Consequently, our paired “postanthesis” samples may not have acted as a negative control for comparison to during-anthesis samples, as we initially intended. Further research is needed to confirm whether the end of male floret VOC emissions may also serve as a cue for pollinator release, which occurs during the afternoon on the second day of the pollination cycle (Prime 1960; Lack and Diaz 1991; Chartier *et al.* 2013).

After comparing male floret transcript expression among populations during anthesis, we did not identify any putative VOC synthases which were unique to Forêt du Gâvre, France. High inter-individual variation in VOC emissions has been widely observed in *A. maculatum* (Gfrerer *et al.* 2021; Szenteczki *et al.* 2021) and this variability is also evident in our transcriptomic dataset. Although our differential expression analyses were unable to identify unique VOC synthases in the Forêt du Gâvre population, targeted investigations into candidate genes underlying key compound classes did provide further insights into species-specific pollinator attraction in *A. maculatum*.

### Candidate transcripts linked to *A. maculatum* VOCs

Most inter-individual variation in *A. maculatum* floral scent centers around variation in the ratio of AAA-derived VOCs to sesquiterpenes (Szenteczki *et al.* 2021). Correspondingly, we identified abundant expression of transcripts putatively involved in the biosynthesis of these 2 compound classes. As predicted, indole synthesis via the TSA subunit was highly expressed in appendix tissue in all studied populations. Some of the TSA transcripts we identified also appear to be homologs of BX1, which more efficiently cleaves indole-3-glycerol phosphate without any interaction with the beta subunit (Frey *et al.* 1997; Kriechbaumer *et al.* 2008). Conversely, TSB was more abundantly expressed in male floret tissue, which would limit indole emissions within the floral trap chamber.

Skatole (3-methylindole) has also been reported in *A. maculatum* VOC emissions (Gfrerer *et al.* 2021; Szenteczki *et al.* 2021), but we did not identify any homologs of indoleacetate decarboxylase in the *A. maculatum* transcriptome, which would produce this compound (Whitehead *et al.* 2008). This may be due to low emissions of skatole by inflorescences sampled in this study; ultimately, the mechanism by which *A. maculatum* produces skatole remains unclear. Prior to this study, it was also unclear whether *A. maculatum* produced *p*-cresol (4-methylphenol) via a pathway homologous to tyrosine degradation in bacteria (Selmer and Andrei 2001; Saito *et al.* 2018), or using 1 or more novel proteins. We were able to identify candidate transcripts (i.e. ISS1 and HPPR homologs) which may catalyze the conversion of tyrosine to *p*-coumaric acid, but were ultimately unable to identify transcripts which could further produce *p*-cresol from *p*-coumaric acid.

Homologs of phenylalanine ammonia-lyase (PAL) and cinnamate 4-hydroxylase (C4H), which produce *p*-coumaric acid from phenylalanine, where also abundantly expressed in the appendix during anthesis. However, downstream proteins producing common FBP VOCs (e.g. eugenol, isoeugenol, benzyl benzoate, benzaldehyde, and phenylacetaldehyde; Muhlemann *et al.* 2014) were largely absent or lowly expressed in both our VOC and transcriptomic datasets. Typically, *p*-coumarate-3-hydroxylase (C3H) would further modify *p*-coumaric acid into precursors for the aforementioned FBP VOCs, but this gene was expressed at relatively low levels in appendix tissue. Interestingly, experimental knock-outs of C3H in *Petunia × hybrida* have demonstrated that downregulation of this gene also leads to unexpected production of *p*-cresol (Kim *et al.* 2019). Given that we observed similar patterns in *A. maculatum*, it appears that flowering plants may have a novel mechanism for producing *p*-cresol involving *p*-coumaric acid, which is distinct from known bacterial proteins such as hydroxyphenylacetate decarboxylase.

While not derived from amino acids, 2-heptanone is another abundant *A. maculatum* VOC with a “fermented” scent. In this study, we were able to identify several methylketone synthase homologs (MKS1 and MKS2), which produce 2-heptanone and related compounds in tomato plants (Ben-Israel *et al.* 2009; Khuat *et al.* 2019). 2-heptanone is known to be attractive to fruit flies (Prokopy *et al.* 1998) and beetles (Torto *et al.* 2007); however, it has not yet been linked with the attraction of specific pollinators of *A. maculatum* (Espíndola *et al.* 2011; Szenteczki *et al.* 2021). While this compound is infrequent in VOC bouquets, it can be a proportionally large component of VOC emissions when it is produced (Szenteczki *et al.* 2021); further research is therefore needed to determine whether this compound is also under selection (e.g. by Drosophilidae).

We also found that DXS activity was elevated in *A. maculatum* appendix tissue during anthesis. An identical expression pattern was also identified in *A. concinnatum*, where it was hypothesized that DXS is the rate-limiting step in monoterpene synthesis (Onda *et al.* 2015). If DXS is also a rate-limiting enzyme in *A. maculatum*, then this may explain the proportionally smaller quantities of monoterpenes in its floral blend, in comparison to other VOC chemical families.

Finally, we identified many putative TPSs in the *A. maculatum* transcriptome, the majority of which appear to produce sesquiterpene VOCs. These include cytochrome P450 enzymes (P450s), which can further modify volatile terpenes through oxidation, methylation, or acylation (Hamberger and Bak 2013). P450s were the first proteins identified in *Arum* appendices (Yáhiel *et al.* 1974), and our transcriptomic data further confirm that a diverse suite of P450s are expressed in *A. maculatum* appendix and male floret tissue during anthesis. Notably, we found that a trimethyltridecatetraene synthase (Cyt P450 92C6) homolog appears to be correlated with the production of at least 1 unnamed *A. maculatum* sesquiterpene (Kovats RI 1681). This unnamed sesquiterpene was the single strongest predictor of *P. grisescens* attraction in a large-scale survey of *A. maculatum* pollinators (Szenteczki *et al.* 2021). Until now, it has not been possible to experimentally test whether this compound alone is attractive to Psychodidae. However, the candidate gene we identified could be useful in future research aiming to produce this compound through heterologous expression in yeasts (Dhandapani *et al.* 2017; Kutyna and Borneman 2018).

Our results also confirm that bicyclogermacrene synthase (Crocoll *et al.* 2010) is consistently and almost exclusively expressed in male floret tissue during anthesis, in accordance with the dominance of this sesquiterpene in *A. maculatum* floral chamber scent (Kite *et al.* 1998). Interestingly, previous studies have demonstrated that 9-methyl germacrene B is a sex pheromone produced by male *Lutzomyia longipalpis* (Psychodidae) to attract females (Hamilton *et al.* 1996; Hamilton *et al.* 1999). Consequently, *A. maculatum* may emit bicyclogermacrene, or a closely related sesquiterpene compound as a as part of their deceptive pollination strategy (i.e. as a pheromone mimic). Such terpene emissions in the floral chamber could stimulate the movement of trapped Psychodidae over the male and female florets, aiding in pollination and pollen dispersal; however, further experiments are needed to test these hypotheses. A recent gas chromatography–electroantennography (GC–EAD; Schiestl and Marion-Poll 2002) study demonstrated that *P. phalaenoides* antennae responded most strongly to *p*-cresol, germacrene D, and several other unnamed mono- and sesquiterpenes (Gfrerer *et al.* 2022); these findings are consistent with our transcriptomic results. However, *P. grisescens* antennal responses were not tested as part of this study. Consequently, the results of our coinertia analyses could be used to formulate hypotheses for future GC–EAD research.

### Male floret-specific TPSs and their influence on pollinator attraction

There is growing evidence to suggest that tissue-specific transcript expression of VOC synthases is a common characteristic of deceptive pollination systems (Wong, Amarasinghe, *et al.* 2017, and references therein), and our results confirm similar patterns in *A. maculatum*. Specifically, our coinertia analyses identified significant covariation between male floret TPS expression, and the communities of Psychodidae pollinators trapped by inflorescences. While the floral scent of *A. maculatum* is often described as dung-like due to abundant emissions of amino acid-derived VOCs, our results suggest that terpene VOCs may be subject to pollinator-mediated selection as well. Recent large-scale ecological studies also support this hypothesis, with sesquiterpene compounds, rather than so-called “dung-mimicking” compounds, being a better predictor of variation in pollinator attraction patterns (Szenteczki *et al.* 2021) and fruit set size (Gfrerer *et al.* 2021) in *A. maculatum*.

Some of the main compounds related to sex- and species-specific attraction in our coinertia analysis included humulene (covarying with female *P. phalaenoides*), and the aforementioned trimethyltridecatetraene synthase homolog, which may produce an unnamed sesquiterpene with a Kovats Retention Index of 1681 (covarying with *P. grisescens*). Notably, these same compounds were highlighted as key predictors of pollinator species trapped by *A. maculatum* inflorescences in a random forest analysis with a larger sample size (Szenteczki *et al.* 2021). Further research is therefore needed in order to assess the importance of male floret VOCs for pollinator attraction in *A. maculatum* and to confirm the true identities of the compounds produced by the candidate TPSs we identified.

## Conclusion

Our data provide a deeper understanding of the relationships between transcript expression, floral scent, and pollinator attraction in *A. maculatum*, and may lead to the identification of new VOC biosynthetic genes in the future. It appears that *A*. *maculatum* inflorescences employ a combination of highly diverse appendix VOCs including 2-heptanone, indole, and *p*-cresol to attract a broad range of coprophilous dipterans to their inflorescences, and specialized sesquiterpene emissions to further lure specific Psychodidae into the floral trap chamber. Male floret-specific VOCs, particularly bicyclogermacrene, may also play a role in retaining pollinators until the pollination cycle is complete. While Kite and colleagues (Kite 1995; Kite *et al.* 1998) separately characterized *A. maculatum* appendix and trap chamber VOC emissions, all subsequent studies analyzed total headspace profiles (i.e. a blend of appendix and trap chamber VOCs). Our results highlight a need for further focused study of VOCs within the trap chamber, given that specific sesquiterpenes appear to be an important aspect of this highly specialized lure-and-trap pollination system.

## Data availability

Supplementary Appendices 1 and 2 contain supporting figures and tables referenced in the main text. Raw Illumina RNA-seq reads have been deposited in the NCBI SRA database under the BioProject Accession PRJNA856436 (https://www.ncbi.nlm.nih.gov/bioproject/PRJNA856436). Additional data, including the files and R code needed to reproduce all analyses and figures, have been archived in the Zenodo repository 6806366 (https://doi.org/10.5281/zenodo.6806365).

Supplemental material is available at *G3* online.

## Supplementary Material

jkac175_Appendix_S1

jkac175_Appendix_S2
