# Supplementary material for: Transcriptomic analysis of deceptively pollinated Arum maculatum (Araceae) reveals association between terpene synthase expression in floral trap chamber and species-specific pollinator attraction
Source: G3 (Bethesda). 2022 Jul 21;12(9):jkac175. doi: 10.1093/g3journal/jkac175 (PMC9434142; doi:10.1093/g3journal/jkac175)
Supplement: jkac175_Appendix_S1 [file jkac175_appendix_s1.docx]

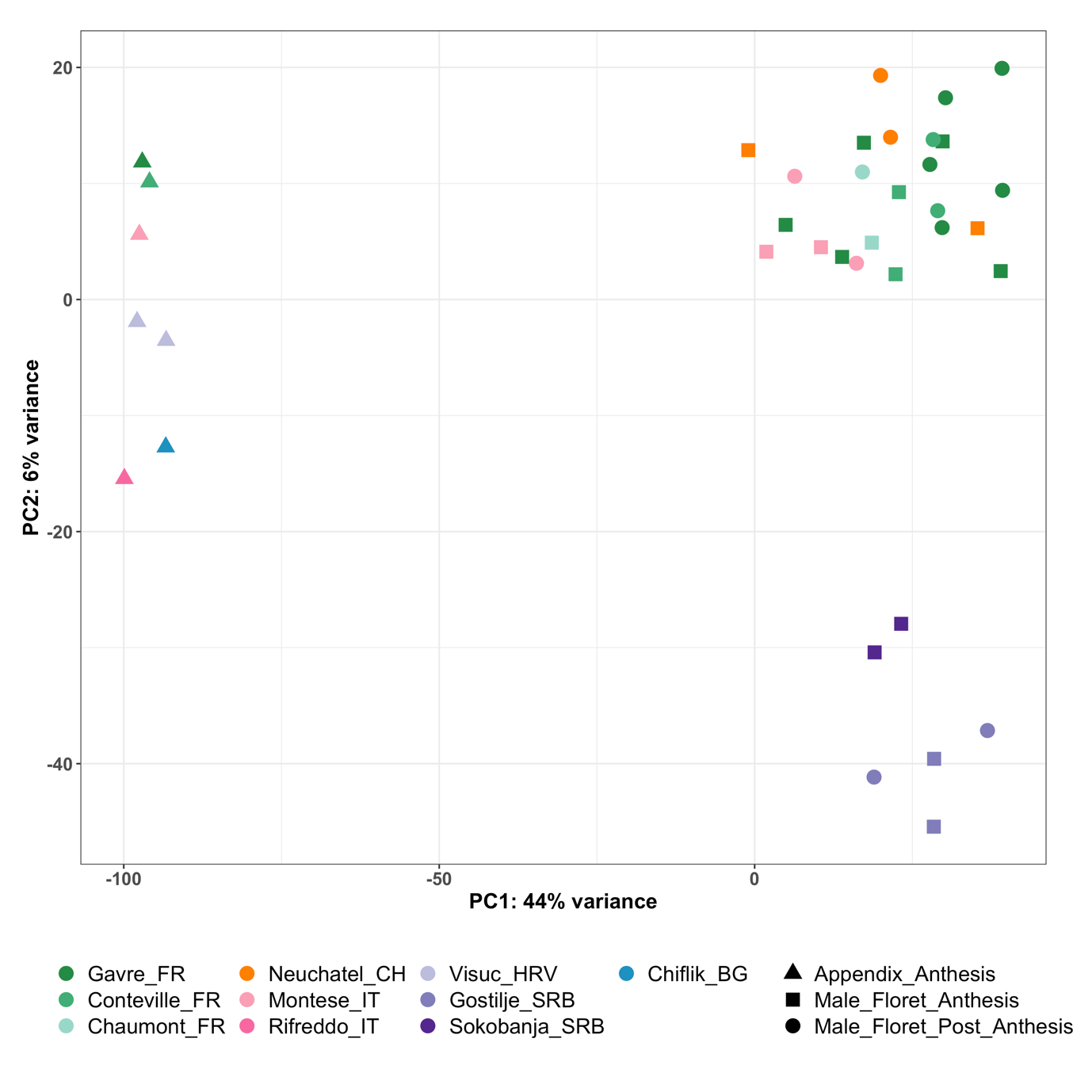


**Figure S1.** Principal components analysis visualizing *Arum maculatum* transcript expression among all populations (denoted by color) and tissue types/sampling times (denoted by shape) in this study. This analysis included all 49’779 transcripts remaining following filtering in DESeq2 (*i.e.* >0.75CPM in at least seven samples).

**
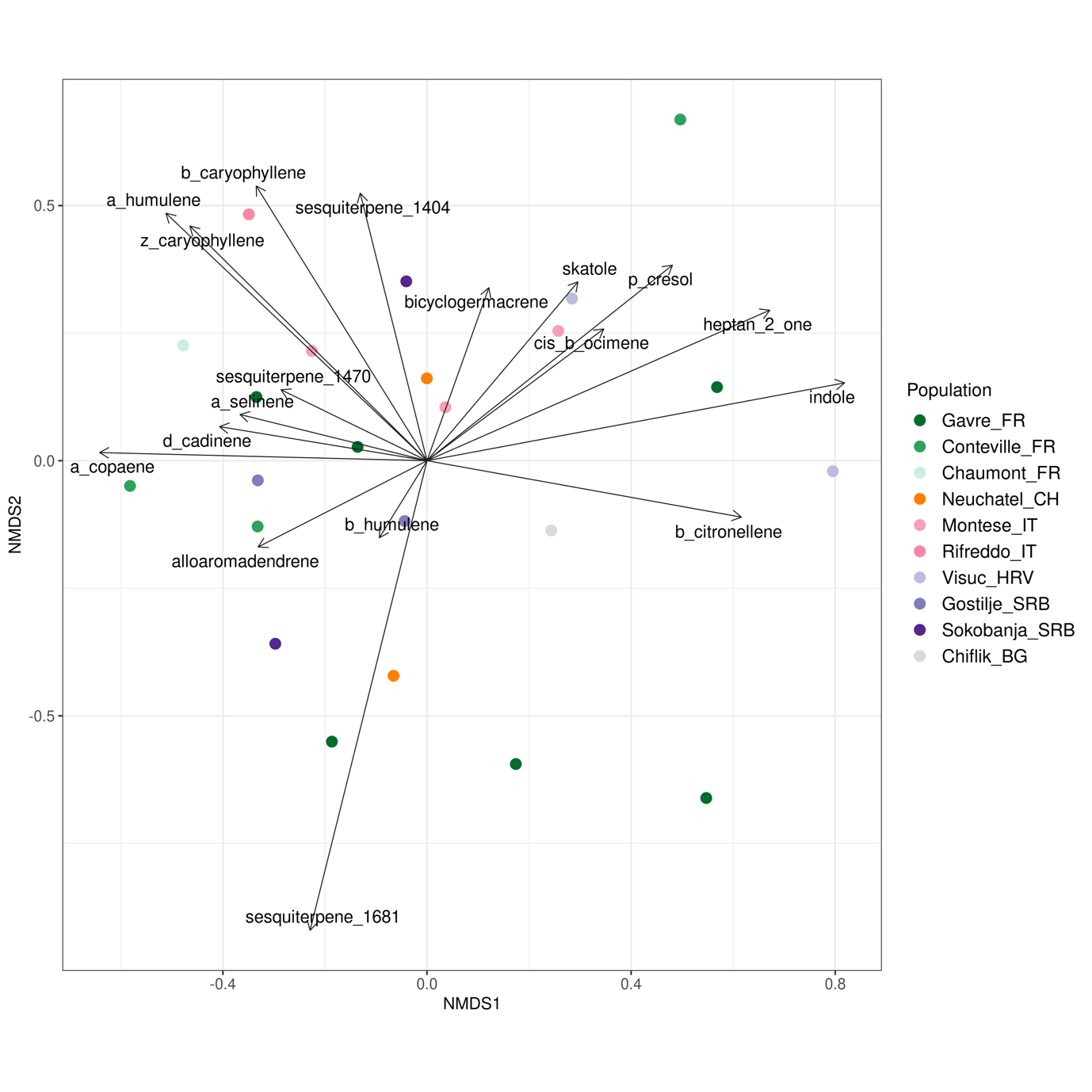
Figure S2.** Nonmetric multidimensional scaling (NMDS; Bray–Curtis dissimilarity, stress = 0.0582) plot of floral scent differentiation among individual *Arum maculatum* inflorescences sequenced as part of this study. Analyses were performed on a matrix of the proportional abundances of all 18 volatile organic compounds identified in *A*. *maculatum* bouquets (see Table S5, below). **Figure S3.** Heatmaps visualizing expression of the methyl-D-erythritol 4-phosphate (MEP; above) and mevalonate pathway (following page) for terpenoid precursor biosynthesis in *Arum ma­­culatum*. Color scale represents the *vst*-transformed mean expression of transcripts across tissue types, populations, and stages of anthesis.

**Figure S3** **(cont.**)

**
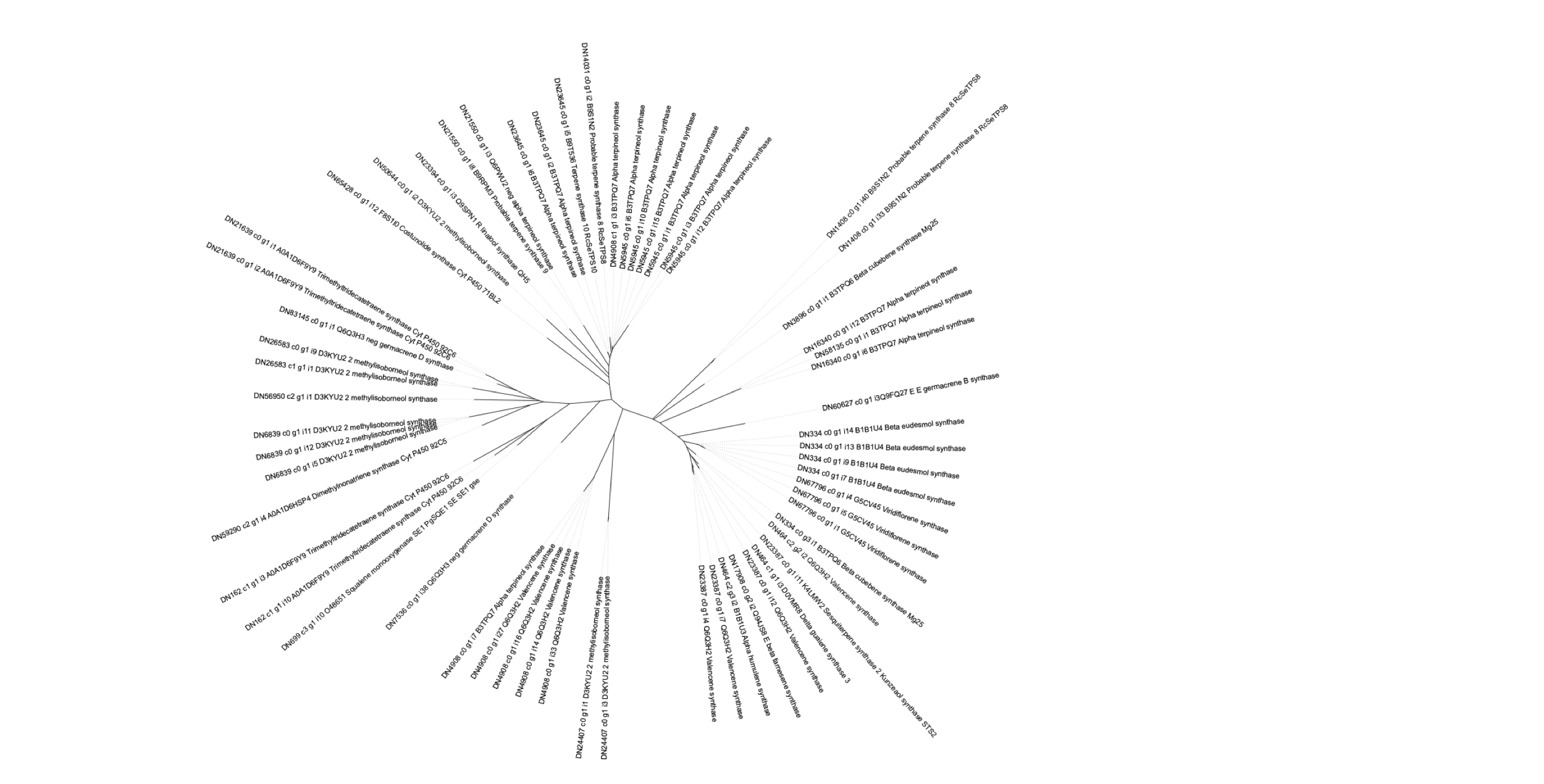
Figure S4**. Unrooted phylogeny of all putative *Arum* *maculatum* terpene synthase isoforms expressed in appendix and male floret tissue during anthesis.

Note: Gene names were derived from automated annotation alone, and should therefore be considered hypotheses.

| Reference sequence (1): sp|D3KYU2|MIBS_STRLS  Colored by: identity |
| --- |
| **1** **[ . . . . : . . .** **80**  1 sp|D3KYU2|MIBS_STRLS **MPDSGSLGPPTSLPEQPPAPPATAPDAPAATVTDRPVTSSVAHFLAGLHPPVTRPSSPPSPSMPPASSNPSSPPSSSMPP**  2 DN26583_c0_g1_i9  **--------------------------------------------MSGVTLAVG----------------PRTEPDRTATT**  3 MQL94539.1 **--------------------------------------------MSGVTLAVG----------------PRTEPDRTMTT**  **81**  **. 1 . . . . : .** **160**  1 sp|D3KYU2|MIBS_STRLS **ASWAPPSPLSPPAPSLPPTSPPATAPETSAATGSDSVVRRVPVGPTGLGTTALSLAR-RQAAVPPDAVPAPSGPSAEGPV**  2 DN26583_c0_g1_i9 **AS----SPMA----KQQQARHPSRQPVVGGVMGSLRVIELQ--------LVAFIMVFSASGLVPLLDL--------AYPA**  3 MQL94539.1  **AS----SPTA----KQQHARHPSRKPVVGGVMGSLRVIELQ--------LVAFIMVFSASGLVPLLDL--------AYPA**  **161**  **. . . 2 . . . .** **240**  1 sp|D3KYU2|MIBS_STRLS **VPGLYH-----HPIPEPDPVRVAEVSRRIKRWAEDEVRLYPE--EWEG-------QFDGFSVGRYMVACHPDAPTVDHLM**  2 DN26583_c0_g1_i9 **LVTLYLLALSRLAFPSAGPVTAAP--REVFE-GGLFFRLYVVVGTLVGLFLPLAYVLGGFARGDEHA----VQSATPHLF**  3 MQL94539.1 **LVTLYLFALSRLAFPSAGPVAAAP--REVFE-GGLFFRLYVVVGTLVGLFLPLAYVLGGFARGDDYT----VRSATPHLF**  **241**  **: . . . . 3 . .** **320**  1 sp|D3KYU2|MIBS_STRLS  **LATRLMVAENAVDDCYCEDHGGSPVGLGGRLLLAHTALDHLHTTAEYAPEWSESLGSDAPRRAYRSAMDHFVRAATPSQA**  2 DN26583_c0_g1_i9 **LLSCQILTENLVTSFGVF---SPPVRALVPLLYT---VRRI----FVIIDWTCDVSFN-----------K----RLPA--**  3 MQL94539.1 **LLSCQILTENLVTSFGVF---SPPVRALVPLLYT---VRRI----FVIIDWIYDVCFN-----------K----TLPA--**  **321**  **. . : . . . . 4** **400**  1 sp|D3KYU2|MIBS_STRLS  **DRYRHDMARLHLGYLAEAAW--AETGHVPEVCEYLAMRQFNNFRPCPTITDTVGGYELPADLHARPDMQRVIALAGNATT**  2 DN26583_c0_g1_i9 **-----------TAPLKL---------------------------------------------------------------**  3 MQL94539.1 **-----------TARLKDVAWLWFGRGLALA--------------------------------------------------**  **401**  **. . . . : . . .** **480**  1 sp|D3KYU2|MIBS_STRLS **IVNDLYSYTKELDSPGRHLNLPVVIAEREHLSDRDAYLKAVEVHNELMHAFEAAAAELAADCPVPAVLRFLRGVAAWVDG**  2 DN26583_c0_g1_i9  **--------------------------------------------------------------------------------**  3 MQL94539.1 **--------------------------------------------------------------------------------**  **481**  **. 5 . . . . ]** **547**  1 sp|D3KYU2|MIBS_STRLS  **NHDWHRTNTYRYSLPDFW-------------------------------------------------**  2 DN26583_c0_g1_i9 **-------------------------------------------------------------------**  3 MQL94539.1 **NLIYFSVNLFAFLLPRFLPRAFERYFKERDEARAKSAEDERAPHSPAAAAAAAGQTETNPREEKKAD** |

**Figure S5**. Multiple sequence alignment of 1) *Streptomyces lasaliensis* 2-methylisoborneol synthase, 2) the TransDecoder predicted peptide sequence of an *Arum maculatum* (Araceae) transcript in our dataset, and 3) an unknown gene identified in the *Colocasia esculenta* (Araceae) genome. While the latter two proteins share 92.5% identity, both share approximately 23% identity with the bacterial 2-methylisoborneol synthase gene.


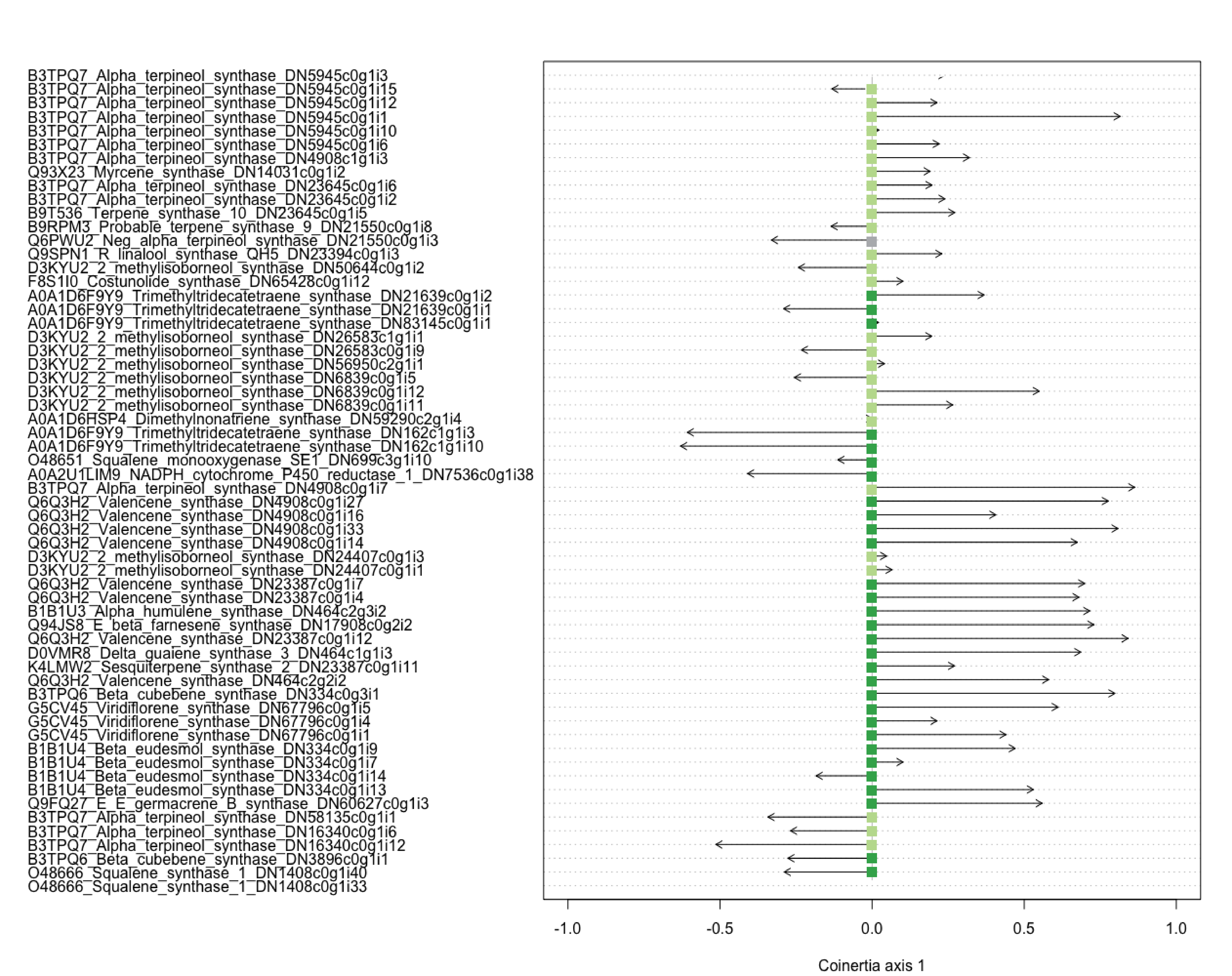

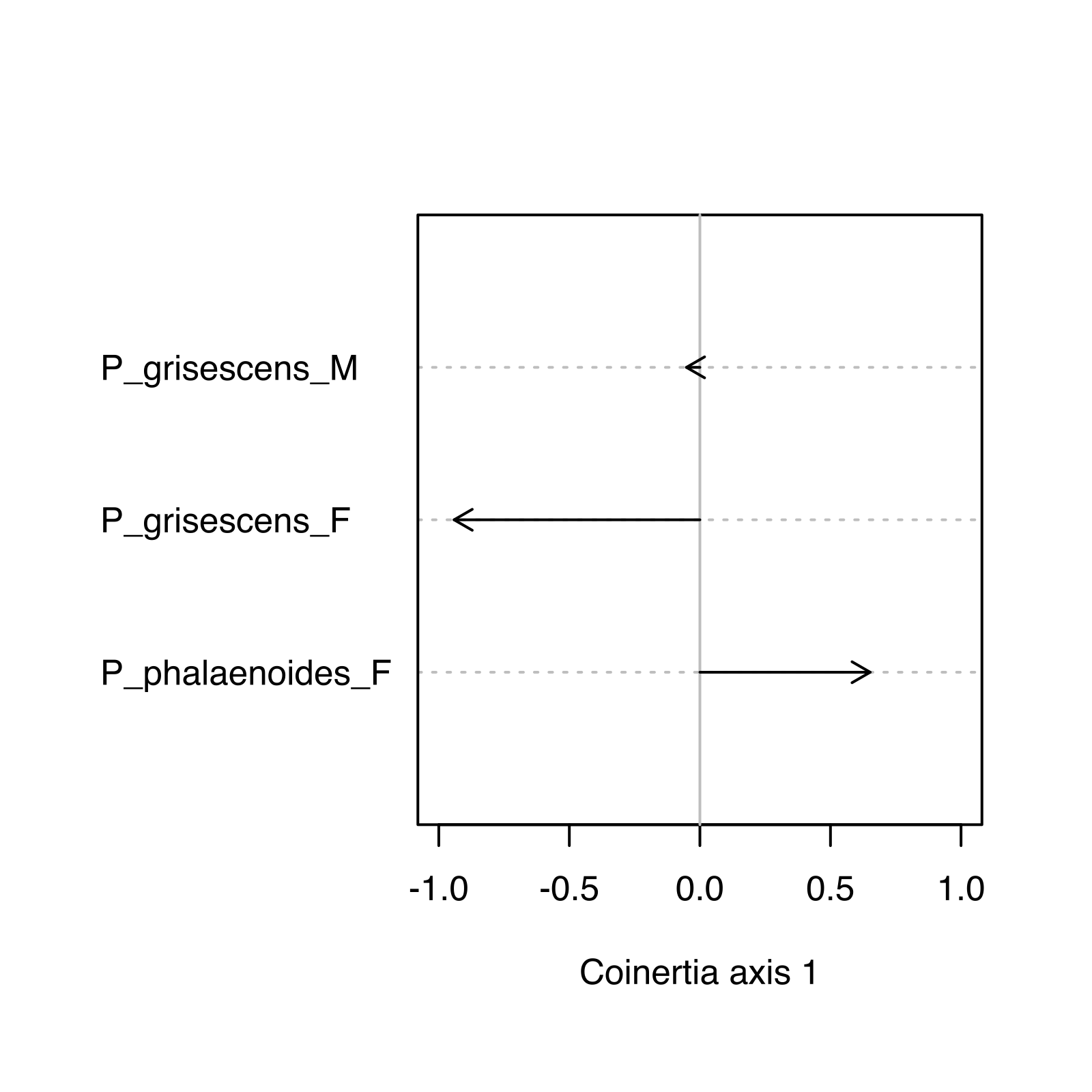


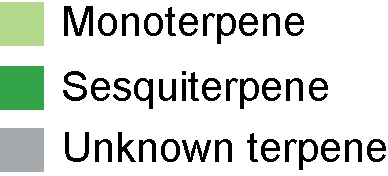


**Figure S6.** Covariation in sex- and species-specific Psychodidae pollinator attraction rates (left plots), and terpene synthase expression in *Arum maculatum* male floret tissue during anthesis (right plots) along the first coinertia axis.


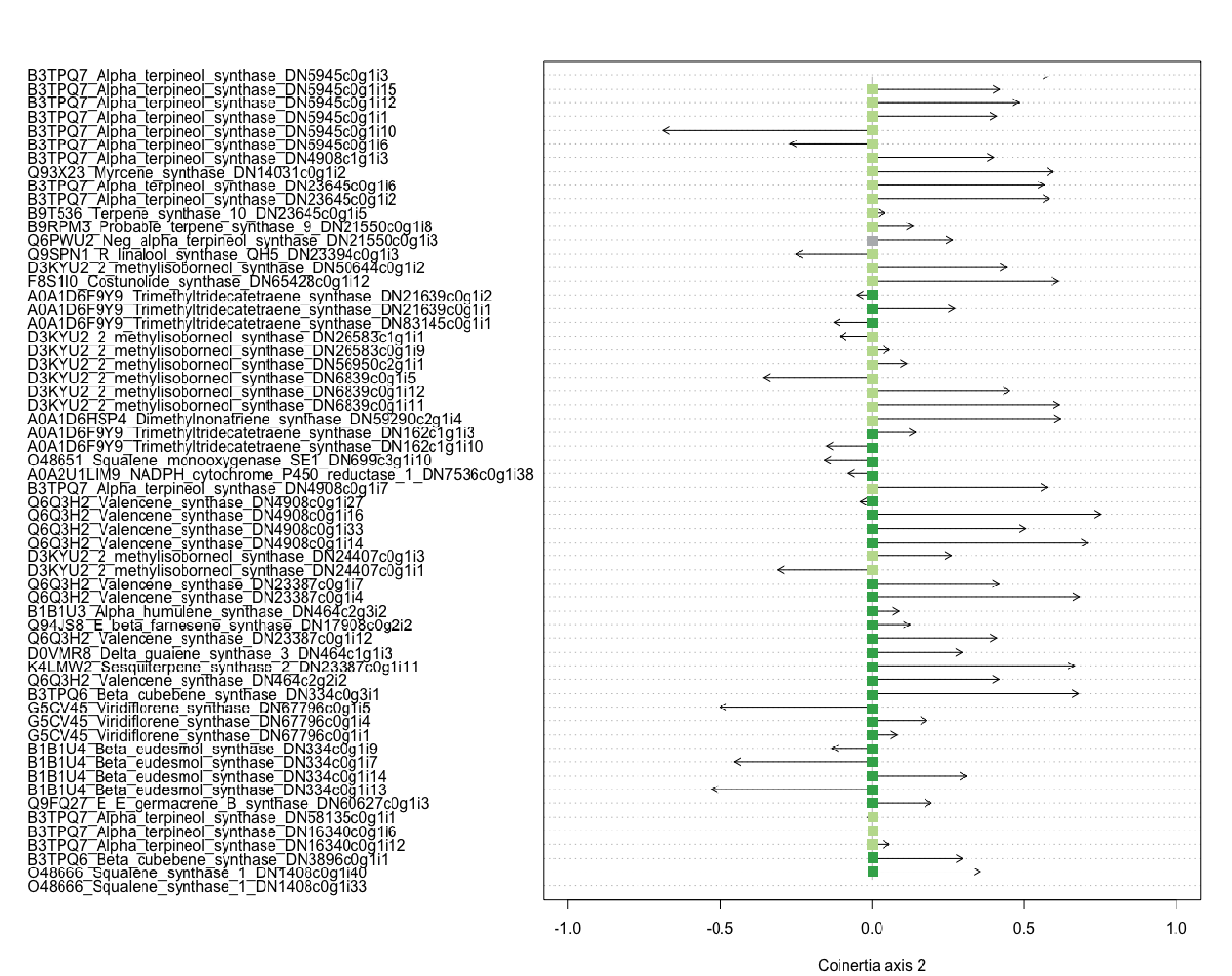

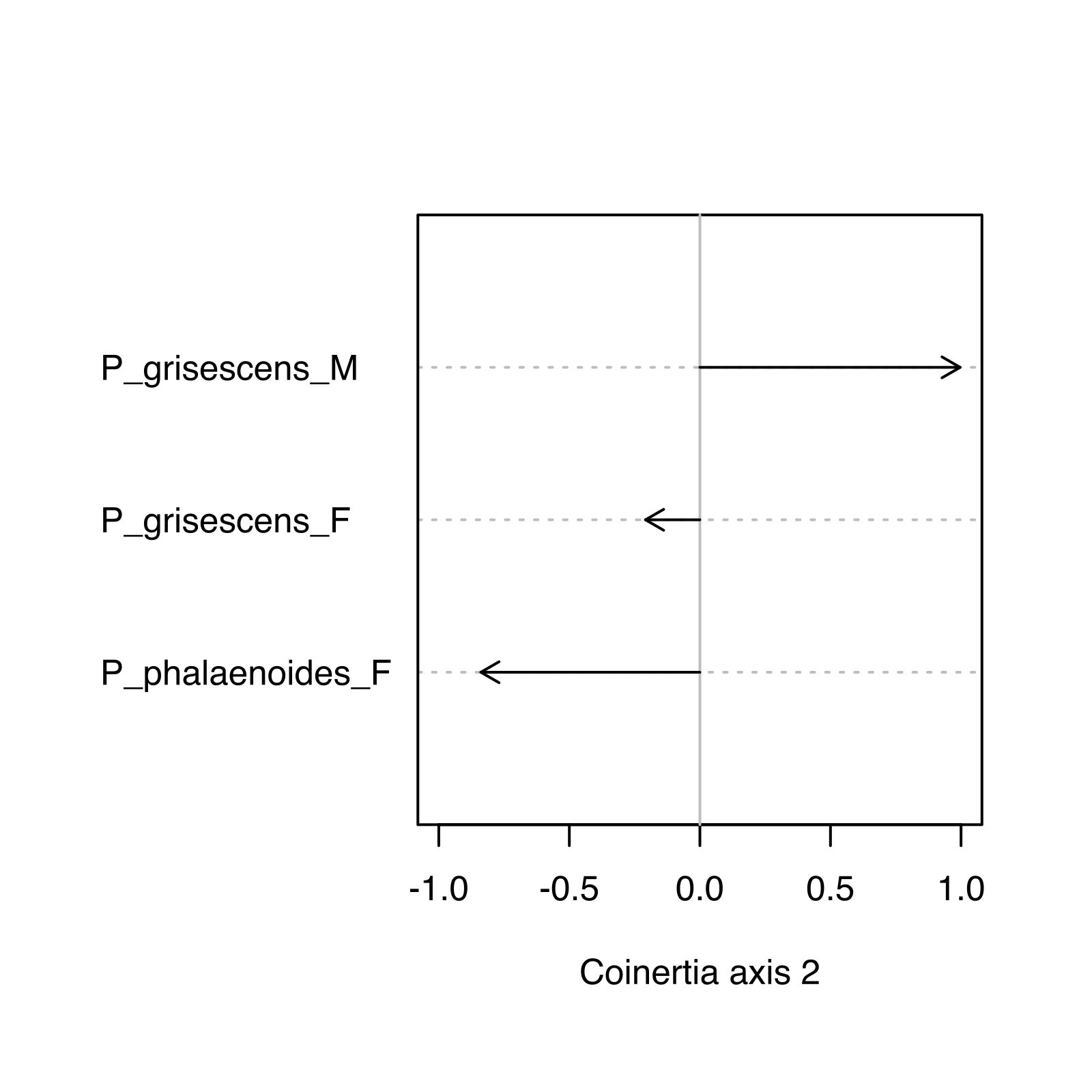
­­


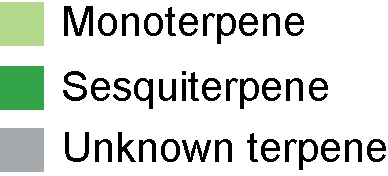


**Figure S6 (cont.)** Covariation in sex- and species-specific Psychodidae pollinator attraction rates (left plots), and terpene synthase expression in *Arum maculatum* male floret tissue during anthesis (right plots) along the second coinertia axis

| **Sample ID** | **Tissue Type** | **Collection Time** | **# reads (raw)** | **# reads (clean)** | **Population** | **Lat.** | **Long.** |
| --- | --- | --- | --- | --- | --- | --- | --- |
| GAV_010 | Appendix | During-Anthesis | 72'325'850 | 66'717'458 | Forêt du Gâvre, France | 47.55066 | -1.86466 |
| CON_016 | Appendix | During-Anthesis | 70'056'626 | 64'913'424 | Conteville, France | 50.73731 | 1.73872 |
| MON_001 | Appendix | During-Anthesis | 62'848'894 | 57'520'886 | Montese, Italy | 44.25523 | 10.98371 |
| RIF_006 | Appendix | During-Anthesis | 59'312'732 | 54'350'550 | Rifreddo, Italy | 40.57235 | 15.82473 |
| VIS_001 | Appendix | During-Anthesis | 65'991'730 | 60'876'804 | Visuc, Croatia | 44.53128 | 15.76134 |
| VIS_003 | Appendix | During-Anthesis | 72'409'196 | 66'825'898 | Visuc, Croatia | 44.53128 | 15.76134 |
| CHI_003 | Appendix | During-Anthesis | 63'427'166 | 58'606'672 | Chiflik, Bulgaria | 42.8130 | 24.52836 |
| GAV_004SA | Male Floret | During-Anthesis | 36'812'088 | 27'933'352 | Forêt du Gâvre, France | 47.55066 | -1.86466 |
| GAV_004CO | Male Floret | Post-Anthesis | 35'212'458 | 29'293'214 | Forêt du Gâvre, France | 47.55066 | -1.86466 |
| GAV_005SA | Male Floret | During-Anthesis | 39'396'020 | 32'399'440 | Forêt du Gâvre, France | 47.55066 | -1.86466 |
| GAV_005CO | Male Floret | Post-Anthesis | 40'842'454 | 33'212'278 | Forêt du Gâvre, France | 47.55066 | -1.86466 |
| GAV_007SA | Male Floret | During-Anthesis | 39'637'246 | 32'386'282 | Forêt du Gâvre, France | 47.55066 | -1.86466 |
| GAV_007CO | Male Floret | Post-Anthesis | 40'504'024 | 32'982'626 | Forêt du Gâvre, France | 47.55066 | -1.86466 |
| GAV_008SA | Male Floret | During-Anthesis | 40'045'110 | 32'600'164 | Forêt du Gâvre, France | 47.55066 | -1.86466 |
| GAV_008CO | Male Floret | Post-Anthesis | 42'181'532 | 33'104'832 | Forêt du Gâvre, France | 47.55066 | -1.86466 |
| GAV_111SA | Male Floret | During-Anthesis | 45'280'290 | 39'923'042 | Forêt du Gâvre, France | 47.55066 | -1.86466 |
| GAV_111CO | Male Floret | Post-Anthesis | 46'879'738 | 41'891'194 | Forêt du Gâvre, France | 47.55066 | -1.86466 |
| CHA_001SA | Male Floret | During-Anthesis | 46'799'844 | 42'716'744 | Chaumont, France | 48.11508 | 5.09475 |
| CHA_001CO | Male Floret | Post-Anthesis | 49'827'838 | 46'503'400 | Chaumont, France | 48.11508 | 5.09475 |
| CON_003SA | Male Floret | During-Anthesis | 39'875'808 | 39'875'808 | Conteville, France | 50.73731 | 1.73872 |
| CON_003CO | Male Floret | Post-Anthesis | 37'195'130 | 29'515'252 | Conteville, France | 50.73731 | 1.73872 |
| CON_005SA | Male Floret | During-Anthesis | 47'294'348 | 41'537'476 | Conteville, France | 50.73731 | 1.73872 |
| CON_005CO | Male Floret | Post-Anthesis | 49'998'560 | 44'422'700 | Conteville, France | 50.73731 | 1.73872 |
| NEU_002SA | Male Floret | During-Anthesis | 37'467'370 | 29'802'078 | Neuchâtel, Switzerland | 47.00043 | 6.9379 |
| NEU_002CO | Male Floret | Post-Anthesis | 42'504'012 | 34'460'112 | Neuchâtel, Switzerland | 47.00043 | 6.9379 |
| NEU_008SA | Male Floret | During-Anthesis | 49'278'902 | 43'503'888 | Neuchâtel, Switzerland | 47.00043 | 6.9379 |
| NEU_008CO | Male Floret | Post-Anthesis | 45'181'684 | 39'358'012 | Neuchâtel, Switzerland | 47.00043 | 6.9379 |
| MON_002SA | Male Floret | During-Anthesis | 44'700'402 | 39'507'032 | Montese, Italy | 44.25523 | 10.98371 |
| MON_002CO | Male Floret | Post-Anthesis | 52'575'366 | 46'259'886 | Montese, Italy | 44.25523 | 10.98371 |
| MON_005SA | Male Floret | During-Anthesis | 45'849'700 | 40'354'964 | Montese, Italy | 44.25523 | 10.98371 |
| MON_005CO | Male Floret | Post-Anthesis | 50'195'542 | 43'370'534 | Montese, Italy | 44.25523 | 10.98371 |
| GOS_001SA | Male Floret | During-Anthesis | 47'615'578 | 42'110'792 | Gostilje, Serbia | 43.65561 | 19.83549 |
| GOS_001CO | Male Floret | Post-Anthesis | 47'897'388 | 41'765'298 | Gostilje, Serbia | 43.65561 | 19.83549 |
| GOS_008SA | Male Floret | During-Anthesis | 42'165'118 | 34'880'800 | Gostilje, Serbia | 43.65561 | 19.83549 |
| GOS_008CO | Male Floret | Post-Anthesis | 41'497'178 | 33'537'420 | Gostilje, Serbia | 43.65561 | 19.83549 |
| SOK_104SA | Male Floret | During-Anthesis | 48'025'822 | 41'973'970 | Sokobanja, Serbia | 43.60373 | 21.88755 |
| SOK_005SA | Male Floret | During-Anthesis | 36'079'684 | 29'352'734 | Sokobanja, Serbia | 43.60373 | 21.88755 |

**Table S1**. Summary of *Arum maculatum* samples collected in this study, and the number of initial (raw) and post-filtering (clean) 150bp paired-end RNA-seq reads generated for each sample.

**Table S2**. Basic assembly quality metrics for our complete *Arum* *maculatum* transcriptome (all contigs). The single Trinity assembly was constructed from all of the appendix and male floret samples listed in Table S1.

| **Method** | **N50 (bp)** | **GC (%)** | **Median length (bp)** | **Average length (bp)** | **Assembled bases** |
| --- | --- | --- | --- | --- | --- |
| Trinity | 1699 | 45.1 | 503 | 949 | 563’420’622 |

**Table S3**. Results from two de-duplication techniques, used to reduce the quantity of redundant isoforms in the raw assembly output by Trinity.

| **Method** | **# of transcripts (raw trinity assembly)** | **# of transcripts (post-filtering output)** | **N50 (bp)** | **GC (%)** | **Average length (bp)** |
| --- | --- | --- | --- | --- | --- |
| EvidentialGene | 593’392 | 195’892 | 1195 | 44.5 | 1140 |
| CD-HIT (95% ID) | 593’392 | 240’758 | 7908 | 47.1 | 925 |

**Table S4**. BUSCO v5.0.0 completeness of the raw Trinity assembly, and two de-duplicated assemblies. EvidentialGene removed a larger quantity of sequences while also better preserving transcriptome completeness, and was therefore used for all subsequent analyses in DESeq2.

| **Method** | **Complete**  **[C]** | **Single-Copy [S]** | **Duplicated**  **[D]** | **Fragmented**  **[F]** | **Missing**  **[M]** |
| --- | --- | --- | --- | --- | --- |
| Trinity (raw) | 95.9% | 6.6% | 89.3% | 2.0% | 2.1% |
| EvidentialGene | 95.6% | 19.5% | 76.1% | 2.2% | 2.2% |
| CD-HIT | 92.2% | 48.3% | 43.9% | 5.2% | 2.6% |

**Table S5**. Average proportional emissions of volatile organic compounds (VOCs) emitted by individual *Arum maculatum* in this study. Additional sample metadata are given in Table S1.

|  | | **Forêt du Gâvre, FR**  **(GAV)** | | | | | | **Conteville, FR**  **(CON)** | | | **Chaumont,**  **FR (CHA)** | **Neuchâtel, CH (NEU)** | |
| --- | --- | --- | --- | --- | --- | --- | --- | --- | --- | --- | --- | --- | --- |
| **VOC** | **RI | ID** | **010** | **004** | **005** | **007** | **008** | **111** | **016** | **003** | **005** | **001** | **002** | **008** |
| 2-heptanone | 891 | 0.0476 | 0 | 0 | 0 | 0 | 0.0227 | 0.0793 | 0 | 0 | 0 | 0 | 0.0004 |
| *β*-citronellene | 943 | 0.0922 | 0 | 0.2170 | 0 | 0 | 0.0033 | 0 | 0 | 0.0022 | 0 | 0.0638 | 0.0055 |
| (*Z*)-*β*-ocimene | 1037 | 0.0144 | 0 | 0 | 0 | 0 | 0 | 0 | 0 | 0 | 0 | 0 | 0.0008 |
| *p*-cresol | 1076 | 0.0606 | 0 | 0 | 0.0327 | 0 | 0 | 0.2411 | 0 | 0 | 0 | 0 | 0 |
| indole | 1289 | 0.5034 | 0.0946 | 0.2218 | 0.0395 | 0.0267 | 0.0406 | 0.2183 | 0 | 0.0107 | 0 | 0.0039 | 0.2660 |
| skatole | 1383 | 0.0002 | 0 | 0 | 0 | 0 | 0 | 0.0006 | 0 | 0 | 0 | 0 | 0.0011 |
| *α*-copaene | 1374 | 0 | 0 | 0 | 0.0660 | 0 | 0.0870 | 0.0159 | 0.0673 | 0.0293 | 0.0396 | 0.0422 | 0.0414 |
| (*Z*)-*β*-caryophyllene | 1405 | 0 | 0 | 0 | 0.0686 | 0.0253 | 0.0484 | 0.0718 | 0.0951 | 0.1044 | 0.0271 | 0 | 0.0154 |
| *(E)-β*-caryophyllene | 1416 | 0.0222 | 0.0651 | 0 | 0 | 0.1480 | 0.0674 | 0.1500 | 0.3467 | 0 | 0.1440 | 0.0206 | 0.1260 |
| α-humulene | 1452 | 0.0213 | 0.0593 | 0 | 0 | 0.1287 | 0.0066 | 0.0473 | 0.0846 | 0.0901 | 0.1122 | 0.0027 | 0.1089 |
| alloaromadendrene | 1459 | 0.0235 | 0 | 0 | 0.1302 | 0 | 0.1954 | 0.0102 | 0 | 0.0558 | 0.0808 | 0.1671 | 0.0180 |
| *β*-humulene | 1473 | 0 | 0 | 0 | 0 | 0 | 0.0326 | 0 | 0 | 0.0868 | 0 | 0.0191 | 0.0021 |
| *α*-selinene | 1491 | 0 | 0 | 0 | 0 | 0 | 0.0390 | 0 | 0 | 0.0149 | 0.4102 | 0.0240 | 0.0552 |
| bicyclogermacrene | 1493 | 0 | 0 | 0 | 0 | 0 | 0.0434 | 0.1654 | 0 | 0.0043 | 0 | 0.0083 | 0.0020 |
| *δ*-cadinene | 1520 | 0 | 0 | 0 | 0.0415 | 0.0821 | 0.0688 | 0 | 0 | 0.0871 | 0.0951 | 0.0221 | 0.0695 |
| Unnamed sesqui. | 1404 | 0.0762 | 0 | 0 | 0 | 0.1330 | 0.1211 | 0 | 0.0212 | 0.0240 | 0.0439 | 0 | 0.0458 |
| Unnamed sesqui. | 1470 | 0 | 0 | 0 | 0 | 0.1189 | 0 | 0 | 0 | 0.0354 | 0 | 0 | 0.1138 |
| Unnamed sesqui. | 1681**^** | 0.1384 | 0.7810 | 0.5612 | 0.6214 | 0.3372 | 0.2238 | 0 | 0.3852 | 0.4550 | 0.0471 | 0.6262 | 0.1282 |

*RI = Kovats retention index, non-polar, HP-5MS ( ^ = RI 1716 in Diaz & Kite 2002)*

**Table S5 (cont.)**

|  | | **Montese, IT**  **(MON)** | | | **Rifreddo, IT (RIF)** | **Visuć, HRV**  **(VIS)** | | **Gostilje, SRB**  **(GOS)** | | **Sokobanja, SRB**  **(SOK)** | | **Chiflik, BG (CHI)** |
| --- | --- | --- | --- | --- | --- | --- | --- | --- | --- | --- | --- | --- |
| **VOC** | **RI | ID** | **001** | **002** | **005** | **006** | **001** | **003** | **001** | **008** | **005** | **104** | **003** |
| 2-heptanone | 891 | 0.1615 | 0 | 0 | 0 | 0.5202 | 0.0911 | 0 | 0 | 0 | 0 | 0 |
| *β*-citronellene | 943 | 0.0544 | 0.0154 | 0.0303 | 0.0199 | 0.1044 | 0.0554 | 0 | 0.0065 | 0.0051 | 0 | 0 |
| (*Z*)-β-ocimene | 1037 | 0 | 0.0028 | 0 | 0 | 0 | 0.0195 | 0 | 0 | 0 | 0 | 0 |
| *p*-cresol | 1076 | 0.0216 | 0.0069 | 0 | 0 | 0 | 0.1493 | 0 | 0 | 0 | 0 | 0.0856 |
| Indole | 1289 | 0.2245 | 0.2631 | 0 | 0.0102 | 0.1572 | 0.2417 | 0.0910 | 0.0007 | 0.0571 | 0 | 0.2703 |
| skatole | 1383 | 0.0025 | 0 | 0 | 0 | 0 | 0.0051 | 0.0024 | 0 | 0 | 0 | 0 |
| *α*-copaene | 1374 | 0.0210 | 0.0302 | 0.0300 | 0.0151 | 0 | 0.0178 | 0.0129 | 0.0626 | 0.0246 | 0.0845 | 0.0135 |
| (*Z*)-*β*-caryophyllene | 1405 | 0.0205 | 0.0165 | 0.0260 | 0.2302 | 0 | 0.0274 | 0 | 0.0087 | 0.0027 | 0 | 0 |
| *(E)-β*-caryophyllene | 1416 | 0.0518 | 0.0833 | 0.0431 | 0.4863 | 0.0817 | 0.0183 | 0.0383 | 0.0554 | 0.0514 | 0.0612 | 0.0210 |
| *α*-humulene | 1452 | 0.0278 | 0.0693 | 0.0461 | 0.0519 | 0 | 0.0354 | 0.0319 | 0.0682 | 0.0416 | 0.0163 | 0.0218 |
| alloaromadendrene | 1459 | 0.0294 | 0.0525 | 0.0736 | 0 | 0 | 0.0132 | 0.0139 | 0.0633 | 0.0321 | 0.1367 | 0.0200 |
| *β*-humulene | 1473 | 0 | 0.0086 | 0 | 0 | 0 | 0 | 0.0034 | 0.0539 | 0.0028 | 0 | 0.2086 |
| *α*-selinene | 1491 | 0.0086 | 0.0136 | 0 | 0.0159 | 0 | 0.0177 | 0.3609 | 0.0559 | 0.0061 | 0.0474 | 0 |
| bicyclogermacrene | 1493 | 0.0152 | 0.0173 | 0 | 0 | 0 | 0 | 0.0039 | 0.0057 | 0.0106 | 0.0308 | 0 |
| δ-cadinene | 1520 | 0 | 0.0695 | 0.1123 | 0 | 0.0458 | 0.0706 | 0.0291 | 0.1766 | 0.0462 | 0.0505 | 0.0203 |
| Unnamed sesqui. | 1404 | 0.2219 | 0.1379 | 0.4402 | 0.0256 | 0 | 0.1808 | 0.0435 | 0.0337 | 0.6572 | 0 | 0.0266 |
| Unnamed sesqui. | 1470 | 0 | 0.0431 | 0 | 0 | 0 | 0 | 0.0043 | 0.1000 | 0.0169 | 0 | 0 |
| Unnamed sesqui. | 1681**^** | 0.1395 | 0.1698 | 0.1985 | 0.1449 | 0.0907 | 0.0568 | 0.3643 | 0.3086 | 0.0456 | 0.5726 | 0.3124 |

*RI = Kovats retention index, non-polar, HP-5MS ( ^ = RI 1716 in Diaz & Kite 2002)*

**Table S6.** Transcripts highly correlated with Hydroxyphenylpyruvate reductase (HPPR) expression in *A. maculatum* appendix tissue during anthesis (Pearson correlation test, *p* < 0.0001).

| **Trinity ID** | ***PCC*** | ***p* value** | **annotation** |
| --- | --- | --- | --- |
| DN4259_c0_g1_i27 | 0.998 | 0.000000171 | P41893 Low molecular weight phosphotyrosine protein phosphatase |
| DN10943_c0_g2_i9 | 0.992 | 0.000010198 | Q10359 Alpha-1,2-galactosyltransferase gmh3 |
| DN11383_c0_g1_i18 | 0.989 | 0.000023743 | Q9FVZ7 Membrane steroid-binding protein 1 |
| DN17083_c0_g1_i4 | 0.988 | 0.000027273 | [unannotated transcript] |
| DN24139_c0_g1_i18 | 0.988 | 0.000029805 | Q9SQT8 Bifunctional 3-dehydroquinate dehydratase/shikimate dehydrogenase |
| DN3154_c0_g1_i24 | -0.985 | 0.000055587 | Q8VZG7 Ribonuclease TUDOR 1 (AtTudor1) |
| DN4046_c2_g1_i6 | -0.984 | 0.000062665 | Q2KIC0 Nuclear speckle splicing regulatory protein 1 |
| DN7988_c5_g1_i11 | -0.983 | 0.000074481 | Q24206 Broad-complex core protein isoform 6 |
| DN9669_c0_g1_i5 | 0.982 | 0.000074962 | Q9SI61 Amidophosphoribosyltransferase 1 |
| DN2090_c20_g1_i2 | -0.982 | 0.000083468 | Q9LDD1 Probable BOI-related E3 ubiquitin-protein ligase 3 |

PCC = Pearson correlation coefficient
